# Practices for Braiding Indigenous Knowledges and Western Sciences for Research and Monitoring of Biodiversity in Canada

**DOI:** 10.1002/ece3.72358

**Published:** 2026-01-22

**Authors:** Ella Bowles, Dominique A. Henri, Jennifer F. Provencher, Steven M. Alexander, Nicola E. Love, Jade Steel, Carmen Chelick, Junaid S. Khan, Jessica J. Taylor, Britney Zacharuk, Alana Wilcox, Oscar Hartman Davies, Deborah McGregor, Susan Chiblow, Steven J. Cooke, Adam T. Ford, Jesse N. Popp

**Affiliations:** ^1^ Environment and Climate Change Canada Gatineau Quebec Canada; ^2^ University of British Columbia Okanagan Kelowna British Columbia Canada; ^3^ Fisheries and Oceans Canada Ottawa Ontario Canada; ^4^ University of Victoria Victoria British Columbia Canada; ^5^ Gift Lake Métis Settlement Big Lakes County Alberta Canada; ^6^ Carleton University Ottawa Ontario Canada; ^7^ University of Oxford Oxford UK; ^8^ Whitefish River First Nation, Anishinabek Nation Birch Island Ontario Canada; ^9^ University of Calgary Calgary Alberta Canada; ^10^ York University Toronto Ontario Canada; ^11^ Garden River First Nation, Anishinabek Nation Garden River Ontario Canada; ^12^ University of Guelph Guelph Ontario Canada; ^13^ Wiikwemkong Unceded Territory, Anishinabek Nation Wikwemikong Ontario Canada

**Keywords:** biodiversity research and monitoring, braiding, Canada, Indigenous knowledges, pillars and priorities, Western sciences

## Abstract

There has been a widespread effort to braid multiple knowledge systems in biodiversity research and monitoring, yet there is further need to consider how to do so. We interviewed Indigenous Peoples and representatives of 12 Indigenous communities, completed a systematic review of biodiversity studies that utilized Indigenous knowledges (IK) and Western sciences (WS) in Canada, and then braided the outcomes of the conversations and literature review to address if, when, and how IK and WS can be brought together for biodiversity research and monitoring in Canada. Overall, there was a great deal of support for, and desire to, braid IK and WS among interview participants. A suite of nine pillars and priorities was identified for doing so from participants' responses. These priorities included: (1) build and foster relationships; (2) IK should guide projects; (3) Indigenous communities should lead projects; (4) IK must be respected equally with WS; (5) embrace reciprocity (focus on people) and (6) embrace responsibility (focus on land) to the land and one another; (7) ensure equal gender and age representation; (8) intergenerational knowledge transfer is important; and (9) language revitalization is critical. The extent to which the pillars and priorities for braiding were reflected in the current literature varied, and we identified indicators that may help project leads choose what to prioritize in design to fulfill the pillars. These indicators included engagement, relevance, governance, and accessibility. The stages of projects at which IK and WS were brought together (i.e., design, data collection, analysis, reporting, and decision‐making), the roles for each IK and WS at various project stages, and the methods for IK collation and WS data collection varied extensively across the literature. This work deepens our understanding of the practices of knowledge braiding in biodiversity research and monitoring in Canada and offers a toolkit for doing so.

## Graphical abstract

Our research article addresses ‘if, when and how’ to bring together Indigenous knowledges (IK) and Western sciences (WS) in practice in biodiversity research and monitoring, though a combination of interviews/sharing circles with Indigenous Peoples, a systematic literature search, and then braiding outcomes from each. We provide nine pillars and priorities for bridging knowledges that come from the interviews/sharing circles, show how those pillars and priorities are reflected in the current literature, and what biodiversity project leads can focus on in order to meet those pillars and priorities (i.e., Indicators for braiding, shown in the Figure associated with this graphical abstract). We end by outlining the roles that each IK and WS can have across the stages of a project (from design to reporting and decision‐making).

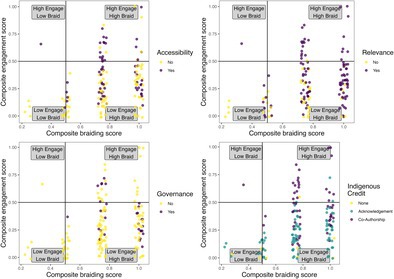

## 1 Introduction

We open this article with guiding words from Indigenous knowledge holders who practice braiding Indigenous knowledges and Western science. Their words reflect the need and desire to braid knowledges and set the table for the rest of the article.

From what I've been told, there's prophecies that we have that we'll come to a point on this planet where we will have to bring all the Peoples and their knowledge together to ensure the survival of humanity. —Susan Chiblow, participant and co‐author

Traditional knowledge is scientific knowledge. —Connecting Guardians workshop participant

For our community, I find that our members were going out and being educated and walking the Western and Traditional paths, having a foot in both worlds. That's what is working for us. So, we are able to bring those things into our stewardship and what we know traditionally, then we have to adapt what Western [knowledge] is teaching and the sciences to suit what we need in our territory. —Connecting Guardians workshop participants

Biodiversity conservation and sustainability efforts led by or conducted in collaboration with Indigenous Peoples, communities, or institutions have different objectives, indicators (ecological, social, and social‐ecological) and outcomes than studies led by a more Western approach. They also yield better outcomes for both the environment and people (e.g., biophysical changes in land use, ecosystem process, or specific aspects of biodiversity (Dawson et al. 2021; Thompson et al. 2020)). Studies that have brought together Indigenous knowledges (IK) and Western science (WS) (Box 1) engage with communities and across generations (Eisner et al. 2012) and ensure that not only perspectives of the political majority are taken into consideration (Mallory et al. 2006). These studies also take a more whole‐ecosystem view, compel continued use of conservation principles and work toward socio‐ecological health (Reid et al. 2021), and produce more comprehensive evidence resulting in more robust policy advice (Housty et al. 2014; IPBES 2019; Tengö et al. 2017), among other benefits.

Working with IK, led or in partnership with Indigenous People, helps enable ethical and inclusive research (Housty et al. 2014) and has been identified as a priority in the United Nations Declaration on the Rights of Indigenous Peoples (UNDRIP) (UN general assembly 2007), articles 18, 26, 29, 31, 32. Bringing together multiple knowledge systems supports biodiversity and the broader goals of reconciliation (Hessami et al. 2021; Parlee et al. 2005; Polfus et al. 2016). To move forward with earnest efforts in reconciliation and effective conservation, a paradigm shift in how cultures, knowledges, and Nations coexist with each other and with animal and plant kin is needed (IPBES 2019; Menzies et al. 2024; *M’sɨt No'kmaq* et al. 2021). Globally, this is occurring, with broad‐scale adoption of UNDRIP (Webb 2024), and there is some tremendous braiding work being done on environmental projects globally (Bulmer et al. 2024; Esquible et al. 2024; Stefanelli et al. 2017; Stern and Humphries 2022). However, there remain problems with the implementation of UNDRIP (Côté et al. 2024) and a lack of knowledge about *how* to braid knowledges for environmental work appropriately/meaningfully (Jones et al. 2024; Mercier and Jackson 2023; Stefanelli et al. 2017).

In the Canadian context, recognizing the need for a paradigm shift in approach to scientific work (Garnett et al. 2018), braiding IK and WS is increasingly being embraced in biodiversity and environmental research, monitoring, and decision‐making (Alexander et al. 2021; Alexander et al. 2019; Brundtland 1987; Provencher et al., n.d.; IPBES 2019; McGregor 2023). In what is now called Canada, Indigenous communities are bringing together IK and WS for research and monitoring when Western approaches can be useful for the problems being addressed (BC Government 2014; CCIRA, n.d.; Council of the Haida Nation 2018; Grant council of the Crees 2011; Okanagan Nation Alliance 2021). Canada has adopted the United Nations Declaration on the Rights of Indigenous Peoples (UNDRIP) through the United Nations Declaration Act (UNDA 2021), and has committed to an action plan to ensure that laws and policies are aligned with UNDRIP principles. Regionally, provinces like British Columbia have developed their own ‘Declaration Act’ to reflect UNDRIP, leading to new initiatives in biodiversity monitoring like the Together For Wildlife Act (Hessami et al. 2021). Importantly, there has been language in Canadian environmental legislation supporting bringing IK and WS together since 1999 (Canadian Environmental Protection Act (CEPA 1999) and Species At Risk Act (SARA 2002)). More recently, the Impact Assessment Agency, Environment and Climate Change Canada (ECCC) and the Department of Fisheries and Oceans (DFO) have all emphasized the need to work with multiple knowledge systems for research and monitoring (DFO 2019a, 2019b; Parks Canada 2019; Migratory Birds Convention Act (MBCA 1994)); and notably via the Indigenous Knowledge Policy Framework (Impact Assessment Agency of Canada, Transport Canada, Canada Energy Regulator, and Fisheries and Oceans Canada 2019).

However, despite tremendous work with bringing IK and WS together over recent years, there is no mechanism discussed for *how* to work with multiple knowledge systems in publicly available Canadian government biodiversity research and monitoring protocols, and dispersed information otherwise (Buxton et al. 2021; Hill et al. 2019). Furthermore, there can be unresolved tensions between IK and WS that prevent them from being brought together to ensure that initiatives move forward in a good way. Example tensions include epistemological differences, legal issues, imbalances in decision‐making power (Eckert et al. 2020; Jones et al. 2024; McGregor 2021), or form and function of information type (Stern and Humphries 2022). A lack of guidance on how to navigate these situations may be part of why IK often gets left out of decision‐making processes and formal assessments. Previous work that we conducted with Anishinabek communities on climate change (Menzies, Bowles, Gallant, et al. 2022) outlined the desire to learn *how* to braid knowledge systems as a priority and recommended that communities take part in the co‐creation of this learning. With this global, federal, regional, and community‐driven desire to work collectively to respectfully braid knowledge systems, communities and governments alike are asking the question, ‘*how do we actually do braiding*’ (Buxton et al. 2021; Jones et al. 2024; Stern and Humphries 2022)? We note that we use the terms ‘bridging’, ‘braiding’, and ‘weaving’ interchangeably in this work (Box 1).

How and in what ways we bring together multiple knowledge systems may depend in part on the purpose of the work (e.g., research, monitoring, management, and decision‐making), along with geography and culture/scale where the work is based (e.g., community, treaty area) (Bowles et al. 2022; Stern and Humphries 2022). Broad frameworks (models) and agreements on relationships (treaties) exist for bringing knowledge systems together that reflect mutually respectful engagement with Indigenous communities and knowledge systems (e.g., *Etuaptmumk* [Two‐Eyed Seeing; Prosper et al. 2011; Youdelis 2016], *SiQ* [Pedersen et al. 2020], *Kaswentha* [Two‐Row Wampum; McGregor 2004], *Gdoo‐naaganinaa* [Dish with One Spoon; Jacobs and Lytwyn 2020], Walking on Two Legs [Dickson‐Hoyle et al. 2022], Ellam Yua et al.'s framework for co‐production of knowledge) (see reviews by Levac et al. 2018; Reid et al. 2021).

These treaties and models are high‐level representations of what the relationship and exchange of knowledge between Nations should look like. They all reflect the importance of ongoing, dynamic relationships as equals, respecting the integrity of each knowledge system and/or group involved. Some of the models provide implementation guidance on aspects of braiding. For example, Pedersen et al. (2020), Yua et al. (2022), David‐Chavez and Gavin (2018), and Ibbett and Brittain (2019) all provide a lot of specific implementation guidance on how to engage respectfully across project stages. And Tengö et al. (2014, 2017) provide a contemporary example of the steps involved in the application of one of these models to a problem or question in such a way that respects the spirit of all the treaties and models, using the Multiple Evidence‐Based Approach (MEBA). MEBA outlines what is needed at five stages of work (mobilize [knowledge], translate [between knowledge systems], negotiate [discrepancies in outcomes form different knowledge systems], synthesize, and apply), but does not provide details about ‘*how*’ to do this, including the roles for each knowledge type at each stage of knowledge gathering and transfer (Buxton et al. 2021). Some researchers have written about case‐specific practices of bringing together knowledge systems and the roles for each knowledge system in so doing (e.g., Gagnon and Berteaux 2009; Huntington et al. 2004; Reid et al. 2021; Riedlinger and Berkes 2001; Thornton and Scheer 2012). Additionally, see Jones et al. (2024) and Stern and Humphries (2022) for reviews of global fisheries and wildlife, respectively. However, further work and guidance is needed to show how these approaches can be generalized to other contexts.

To help fill the ‘details’ gap in the question ‘*how* to braid’, some of the authors of this article, together with others, have completed a series of systematic maps that present a comprehensive survey of the peer‐reviewed and gray literature for cases bringing together IK and WS in environmental research, monitoring, and decision‐making in Canada (Alexander et al. 2021, 2019; Henri et al. 2021; Provencher et al., n.d.). These systematic maps reported the IK and WS methods used to collate knowledge and collect data, methodology for braiding, species or ecosystem scale of the studies, bibliographic information, and demographics of Indigenous communities involved in the studies. While these previous works mapped the landscape of weaving examples, the next step is to look at what the practice of braiding looked like across project stages, including design, data collection, analysis, reporting, and decision‐making. For example, does braiding occur at every stage of a project? What roles can IK and WS have at each stage of a project? Are there patterns that we can find in how IK and WS are brought together in the literature that can help us design future work?

Additionally, there has been a substantial power dynamic in peer‐reviewed studies, where peer‐reviewed studies have been led by Western scientists for the most part, and have been conducted in ways that do not necessarily reflect Indigenous values and ideas and are harmful to Indigenous communities (Jones et al. 2024; Reid et al. 2024; Stefanelli et al. 2017). Indigenous Peoples braid knowledge systems every day by living in a colonized country and having formal education largely within the Western system. There is therefore a considerable amount of information about if/when and how knowledge systems ought to be braided for research, monitoring, and management within Indigenous communities that researchers and decision‐makers are not aware of. The goal of this article is to provide guidance on if/when and how to braid IK and WS for biodiversity research and monitoring, with guidance coming from Indigenous Peoples in Canada.

### 1.1 Objectives

Building upon the relationships and work we completed with communities of the Anishinabek Nation and other First Nation partners (Gallant et al. 2020; Patterson et al. 2020), together with the systematic map dataset introduced above (Alexander et al. 2021, 2019; Provencher et al., n.d.), we did the following. First, through interviews with Indigenous Peoples, we aimed to outline if/when and how IK and WS should be braided for biodiversity research and monitoring. Second, we explored how best practices for braiding IK and WS shared by interview/sharing circle participants were reflected in the literature and whether there were indicators or specific practices that study leads could use to guide their work. Lastly, we provided a catalog of the roles that IK and WS can have across project stages (design, data collection, analysis, reporting, and decision‐making) and the methods used for collating knowledge or collecting data. We took a Two‐Eyed Seeing approach (Bartlett et al. 2012) throughout this work, with both Indigenous and non‐Indigenous researchers on our authorship team, and working in collaboration with Indigenous partners at all stages of the work in order to engage in knowledge braiding practices as we undertook this study.

## 2 Methods

### 2.1 Positionality

We the authors are both Indigenous Peoples (some of whom have shared their Nations in the author list) and non‐Indigenous Peoples who strive to be allies of Indigenous Peoples. As a group, we arrived at the work from different countries (Canada and the United Kingdom), territories (Treaty, Unceded, and Métis territories), and backgrounds (Indigenous knowledge keepers, social scientists, life scientists, physical scientists, students, researchers, and government employees), with the aim of bringing together our collective strengths to this research. We acknowledge, with gratitude, the multiple current and traditional territories and treaty lands where we work. Together, the team felt the challenge and responsibility to do this work respectfully. Our intent was to uplift the voices of Indigenous Peoples and to provide a toolkit for bringing IK and WS together for researchers and project leads in all levels of industry, government, and academia. We use the term toolkit to imply that practitioners can apply the collection of values, approaches, and methods that we highlight. We hope this toolkit will help human societies live more respectfully with one another and in harmony with our non‐human kin, enabling all to thrive.

### 2.2 Knowledge‐Sharing Circles and Individual Interviews

In order to ask Indigenous Peoples about if/when and how to braid IK and WS, we obtained university ethics approval from the University of British Columbia Okanagan (H19‐01453), Mount Allison University (protocol #102582), and the University of Guelph (REB #20–10‐014). The interview team (EB, AM, CC, JS) invited communities to participate in this research based on prior participation in earlier work (November 2019 workshop (Menzies, Bowles, Gallant, et al. 2022), where this project was initially proposed), and also personal connections who had relevant experience or were referred to us, followed by generic invitations sent to other communities via web searches for contact information. For the web searches, we searched the web for contact information for all Indigenous Nations and then selectively invited communities to help provide more consistent geographic and culture‐group representation. We sent invitations to 49 communities or Tribes and to five larger governing bodies (e.g., Métis Nation of Ontario, Manitoba Métis Federation), either to their lands manager or other individuals who we knew or to whom we were referred, or to generic inboxes (for web searches). Beyond individuals who participated, an additional 11 of the groups we contacted expressed interest in the project, but either communication was dropped, timelines for internal applications were incongruent with ours, or community capacity was overwhelmed due to the COVID‐19 pandemic. Unfortunately, there was insufficient time to send invitations to Inuit communities and allow for a reasonable amount of time to make logistical arrangements due to our chain referral approach to invitations combined with external project timeline constraints, and thus no Inuit communities were invited. Ideally, this could be rectified in the future.

Initially, we invited communities to invite one Elder, one youth (aged 19–30), and one representative from a lands department (or equivalent) for one‐on‐one virtual semi‐directed interviews (virtual due to COVID‐19 restrictions). We also encouraged gender balance through our initial correspondence. Several communities did not feel comfortable with individual interviews and instead preferred to have sharing circles or to be interviewed with one other person (Arsenault et al. 2018). Therefore, interviews included anywhere between one and 12 participants. In addition, some interviewees explicitly stated that they were representing their own views and not speaking for their community, Nation, or Tribe. We (the research team) invited individuals and their communities based on their experience with the subjects of our interviews, which we had discussed with our community contacts in advance. We sent honoraria and tobacco to each participant as was appropriate or desired by the community and/or interviewee ($100 to each Elder and $50 to each lands representative and youth), as well as one tablet (Galaxy Tablet A8) per community as communities desired. Tablets were sent initially to facilitate connectivity due to the need to have virtual interviews because of COVID‐19 safety protocols; however, they were ultimately not usable for this purpose.

**FIGURE 1:**
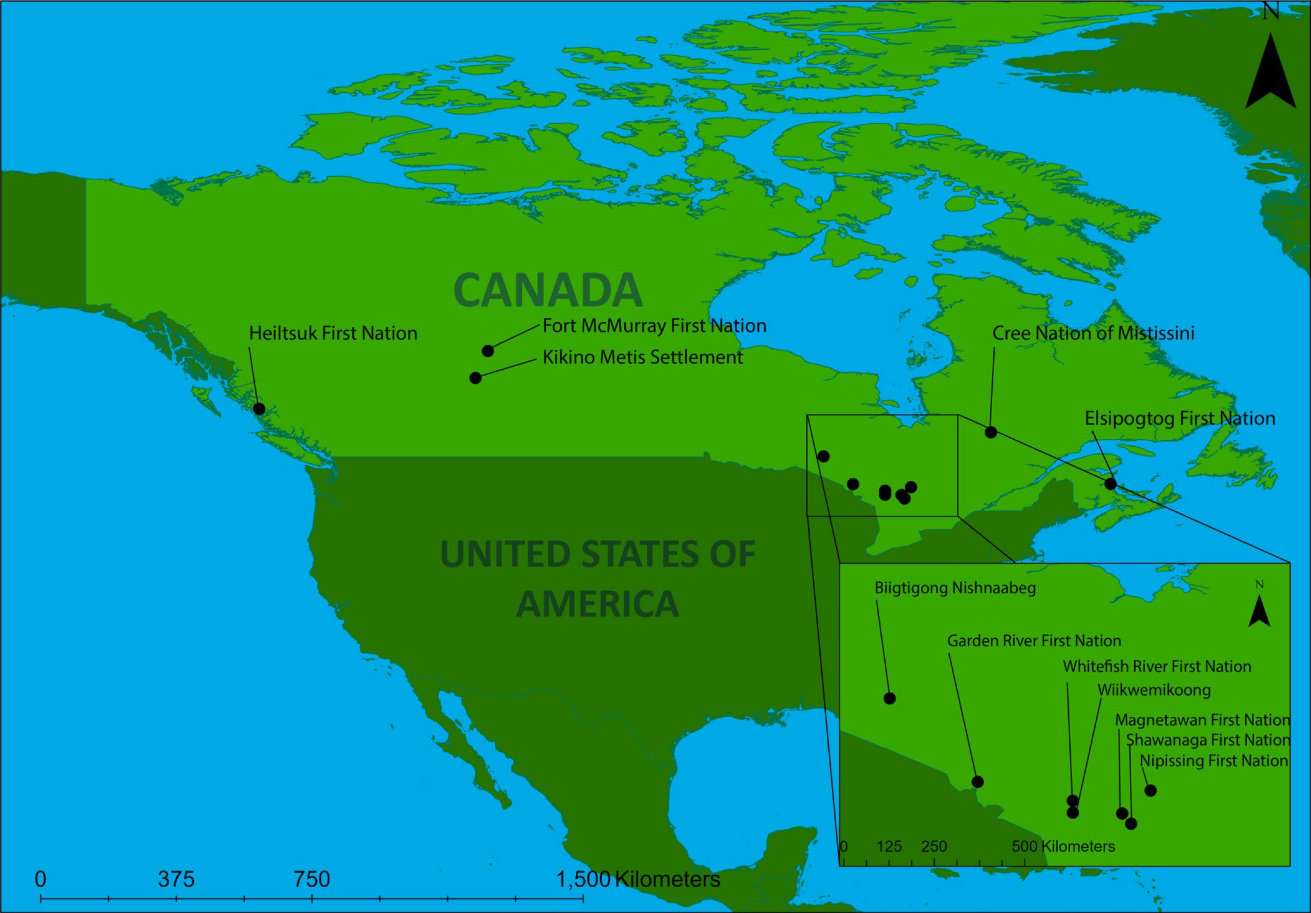
The 12 Indigenous communities across what is now called Canada where the 40 interview/sharing circle participants are members (Bowles, Khan, et al. 2023; Bowles, Menzies, et al. 2023).

We held either semi‐directed individual interviews or sharing circles with members of 12 Indigenous communities across what is now known as Canada, between November 2020 and May 2021 (BC—Haíɫzaqv (Heiltsuk) Nation; AB—Kikino Métis Settlement, Fort McMurray First Nation; ON—Magnetawan First Nation, Garden River First Nation, Wiikwemkoong Unceded Territory, Shawanaga First Nation, Biigtigong Nishnaabeg, Whitefish River First Nation, Nipissing First Nation; QC—Cree Nation of Mistissini; NB—Elsipogtog First Nation) (Figure 1). These communities span several culture groups: Haíɫzaqv, Métis, Cree/Chipewyan, Anishinabek, James Bay Cree, and Mi'kmaw. There were 40 participants in total, with Indigenous community members (38 people) or non‐Indigenous community land managers (2 people) spanning 25 interviews and two sharing circles.

Interviews and sharing circles took anywhere from one to two and a half hours, were recorded on Zoom (saved initially on interviewers' hard drive, and then transferred to EB hard drive), and transcribed by GoTranscript (https://gotranscript.com/). Questions were sent to communities in advance (Supporting Information). In the invitation letter, we offered for translators to be present for interviews, which would include translating consent and all related documents. However, there were no requests for translation; thus, interviews were conducted in English and neither interview materials nor subsequent reports were provided in Indigenous languages. Interviews were anonymized unless participants asked to be identified by name. Free, prior, and informed consent was obtained prior to each interview, and participants were informed that they could withdraw their consent, not answer questions, or discontinue interviews at any time without implications for withdrawal. Long‐term control of data will remain with participants. In the consent letter, we said that audio and video records as well as transcripts would be stored with the First Nation that interviewees belong to for an indefinite period of time, and they would be stored with university‐based researchers (University of Guelph or University of British Columbia) for five years after the completion of the project, after which point they will be deleted from researchers' hard drives. Interview questions were asked of one person in one community to test the questionnaire; questions were revised based on the feedback obtained, and thus responses from the pilot interview were not included in this article, and they were not included in the total number of participants. In order to appreciate where participants were coming from in their answer to our questions about if/when and how to braid knowledges, we asked numerous introductory questions about their experience with caring for the land, how people outside of their communities could help care for the land, and what each IK and WS meant to them (Table S1).

**FIGURE 2:**
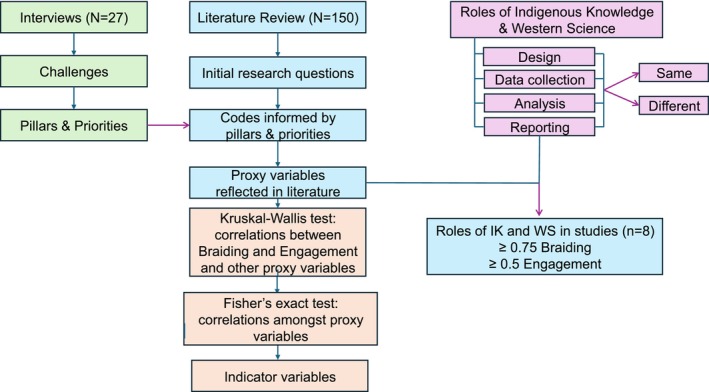
Schematic summarizing how each component of the methods relates to each other. The proportions given for Braiding and Engagement are the composite score values we required for the small subset of studies for which we visualized roles for each IK and WS across all project stages (shown in Figure 5). Braiding is the extent to which IK and WS have been brought together, and Engagement is the extent of collaboration between researchers and Indigenous communities in a given study. The composite score encompasses all project stages such that each study has a single composite score for each variable. For example, a study could have a composite score of 0.5 for engagement and 0.75 for Braiding (See Figure 3).

Thematic analysis using inductive and deductive coding was completed on anonymized transcripts (Saldana 2021) using NVivo (V.12) software (Lumivero 2018) (Figure 2). Every interview transcript was first read and corrected by a single coder (JSK) and then coded with a pre‐determined set of codes, with subsequent codes added as needed (by JSK and EB). Questions and responses discussed herein are a subset of a larger set of questions, which addressed several themes (Bowles, Khan, et al. 2023; Bowles, Menzies, et al. 2023; Menzies et al. 2024). As interviews were coded, often themes from one set of questions emerged in another. In these situations, coders and report authors discussed where best to place that content so that as many perspectives as possible were shared, and duplication of responses was minimized. We followed up with participants to confirm spellings and interpretation of information where needed. Additionally, some quotes included here were edited for clarity. Outcomes of our thematic coding were shared with participants in a knowledge‐sharing event in April 2022, as well as by sharing copies of the reports generated, and, when requested, the recording of the event. Attendance at the knowledge‐sharing event was low, and few participants sent us feedback on the reports, but verbal and written support for outcomes was strong among those who attended and who sent in feedback on the written reports and the recording of the event. This article will also be sent to participants.

The pre‐determined codes were closely matched to the central themes of questions (e.g., braiding IK and WS), while the inductive codes were much more diverse (e.g., power dynamics). Key inductive themes that we present here are how participants defined Indigenous and Western knowledges, what each meant to them, the roles that they felt each should have in environmental monitoring and management, and then several aspects of process and key considerations for if, when, and how to bridge IK and WS. We were ultimately able to summarize a list of challenges and then solutions for those challenges for bridging IK and WS. We term those solutions ‘pillars and priorities’.

### 2.3 Systematic Literature Review and Coding Completed for This Study

The literature included in the database that we analyzed drew from three systematic maps; these maps identified and characterized studies that brought together IK and WS in coastal and marine (to January 2019, Alexander et al. 2019), freshwater (to January 2019, Alexander et al. 2021), and terrestrial (to December 2020, Henri et al. 2021; Provencher et al., n.d.) ecosystems biodiversity research, monitoring, and management in Canada. Systematic mapping is an evidence synthesis method that maximizes transparency and identifies all relevant literature (Haddaway et al. 2017; Haddaway and Westgate 2019). The systematic maps were developed in accordance with the collaboration for Evidence Synthesis Guidelines (Pullin et al. 2018) and Reporting Standards for Systematic Evidence Syntheses in Environmental Research (ROSES) (Haddaway et al. 2018). Details of the search strategy, screening, and coding of papers for the systematic maps are available in Alexander et al. (2019) and Henri et al. (2021). As can be seen in the protocols for the maps, all three had similar objectives and decisions surrounding keyword search strings (e.g., within Canada, regarding biodiversity, types of ecosystem keywords). Upon reviewing all the literature, it was clear that articles focused on braiding knowledge systems in environmental research and monitoring were constructed differently than the ones discussing braiding in environmental management. As such, the two article types (i.e., research and monitoring and management articles) could not be coded in the same way for all metrics. We chose to focus on ‘research and monitoring’ rather than ‘management’ papers, as they are more predictable in structure and content and produce the foundational work for management decisions (*n* = 150 studies across all three systematic maps).

Broadly, for each study we coded bibliographic information, age, and gender of Indigenous knowledge holders involved, species and ecosystem focus of the study, whether Indigenous values were discussed, if and how both IK and WS were utilized at each project stage (i.e., roles for each IK and WS at design, data collection, analysis, reporting and decision‐making), extent of engagement with Indigenous communities at each project stage, and various research quality indicators. We determined what to code for roles for each IK and WS by reading a subset of ~50 articles and determining what these studies had included for roles for each IK and WS. Cleaning and analysis of literature data were done in R (version 4.2.0 (R Core Team 2022)). Note that some of the above information was retroactively coded for the freshwater and coastal marine case studies to meet the goals of the present study; these additional questions had already been coded for terrestrial map literature (See Table S2 for full list of coded questions).

### 2.4 Braiding Indigenous Knowledges and Western Science: Identification of Patterns and Indicators for Braiding IK and WS

We assessed how best practices or pillars and priorities for braiding that were shared by participants during the interviews were reflected in the literature. We aimed to understand patterns in how braiding was done in the literature that could help guide project leads, researchers, and policy makers when they design and conduct projects; that is, we were looking for guidance on the best process for braiding. There were three major pieces to doing so. The first step was to identify which coded information from the systematic map best represented each of the pillars and priorities; we called these codes proxy variables (Table 2). We could then visualize how the pillars and priorities were distributed in the literature (step two). Then, the third step was to test for correlations between the proxy variables to help us understand whether there were associations that could help explain when and why braiding was done with respect to the pillars and priorities. For example, did high engagement between researchers and communities usually mean that braiding occurred at many project stages, or could there also be low engagement and high braiding? Or, if studies coded positively for Governance, did they also correlate highly for braiding?

Step 1: First, we found the coding questions that best aligned with the pillars and priorities identified by participants (Table 2). The terrestrial systematic map coding and interviews were conducted concurrently, and we developed some coding questions for the systematic map that aligned with what we thought may be needed to understand practices for braiding IK and WS; however, we did some re‐coding of literature once we began analysis for this work so that we had coded information for more of the pillars and priorities. As can be seen in Table 2, oftentimes multiple coding questions aligned with a single pillar and priority (e.g., three different demographics of knowledge holders questions aligned with the pillar and priority equal gender and age representation). For ease of working with the coding questions, we gave them each a variable name, which we call proxy variables (Table 2); for example, the information from “Is the study built on a previous relationship or collaboration with an Indigenous community?” was coded as “previous_relationship”.

Step 2: We visualized the distribution of pillars and priorities in the literature by creating composite scores for the proxy variables following a method developed by Littlechild et al. (*unpublished*) and first published by Rathwell et al. (2025). Composite scores aggregated stage‐specific data into a normalized score. We calculated each composite score in two general steps: normalization and aggregation while accounting for missing data. To normalize the scores for each variable, the values within each project stage were divided by the maximum possible value for each metric (one for braiding and four for engagement, etcetera) in order to ensure the values for each metric within each study would not exceed a sum of one. The normalized values were then summed and divided by the total number of project stages with data present within each study. We treated missing values as zeros in the sum across the project stages but discounted them in the denominator to ensure missing values did not falsely inflate the composite value.

Composite braiding and engagement scores were univariate. However, as mentioned above and shown in the groupings of proxy variables in Table 2, most of the pillars and priorities were best represented by several of the proxy variables; thus, initially we combined all the proxy variables that represented a single pillar and priority into composite scores. Therefore, composite scores, like Quality, combined different types of data—like Accessibility, Relevance, and Indigenous credit—into a single metric while also aggregating across project stages within each variable (i.e., multiple variables combined into one). Unsurprisingly, the trends underlying the multi‐variate composite scores were obscured by the diverse indicators they encompassed; therefore, we continued analysis with the separated proxy variables and the single‐variable composites Braiding and Engagement (*n* = 17 variables) (Table 2). We then visualized the studies in the Braiding: Engagement space.

**TABLE 1:** Icons representing the different roles that Indigenous knowledges and Western science had in the case studies (*n* = 150), and the description of how they were brought together (i.e., utilized to address the same question/thing, different question/thing, both the same and different, or not braided at all). Note that the color palettes are distinct for each IK and WS, and orange and gray have generally been used to reflect process (e.g., relationship between roles, questions, percentage, or time).

Pictoral representation	Role for each IK and WS or desciption of braiding	Project stage(s) where coded
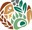	Indigenous knowledges; elements of this icon utilized in all icons where IK and WS are shown together	all
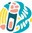	Western science; elements of this icon utillized in all icons where IK and WS are shown together	all
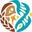	IK and WS used for the same things	Design; Data collection; Analysis; Reporting and decision making
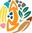	IK and WS used for different things	Design; Data collection; Analysis
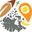	Use IK as local scale expertise	Design; Data collection
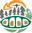	IK as a source of historical/baseline information	Design; Data collection
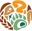	IK used in formulating research questions and hypotheses	Design
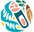	Western science informing IK methods	Design; Data collection
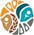	No weaving practices ‐ only IK or WS engaged in design(data collection)(analysis)(reporting and decision making)	Design; Data collection; Analysis; Reporting and decision making
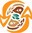	IK used in inferring impacts/changes (i.e., mechanisms‐ for example for population change)	Analysis
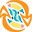	WS used in inferring impacts/changes (i.e., mechanisms‐ for example for population change)	Analysis
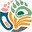	IK is discussed within the existing WS literature or vice versa	Analysis
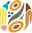	IK and WS are assigned percentage weights for decision‐making	Analysis
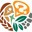	IK supporting identification of further research questions and/or management recommendations	Reporting and decision making
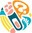	WS supporting identification of further research questions and/or management recommendations	Reporting and decision making

*Note:* The base IK and WS icons contain elements that have then been used individually or in combination in the remaining icons. Both the color and icon elements are used to reflect which knowledge type is being represented in a given icon. These icons were co‐developed with Vincent designs Inc.

Step 3: The next step was to elucidate what correlations existed that could help explain braiding with respect to the pillars and priorities shared by participants, i.e., what pillars and priorities are present when there is braiding at many or few project stages? First, we tested whether Braiding and Engagement were correlated, and which proxy variables (i.e., pillars and priorities) were correlated with Braiding and Engagement scores using Kruskal–Wallis (K–W) tests (Kruskal and Wallis 1952). K–W tests are conservative but robust to continuous and discrete binary and ternary numeric variables. Second, we used Fisher's Exact tests (Fisher 1958) to identify which proxy variables associated with Engagement and Braiding were also correlated with each other. We considered the Fisher's Exact test as a post hoc test, as the proxy variables were decided after we had visualized the data. Third, we grouped variables based on which proxy variables were correlated with each other and found one ‘indicator’ variable, such that when that single variable was present in a particular study, the other proxy variables in that group were usually present in that study as well. Grouping correlated variables allowed us to avoid replicating patterns or overexpressing trends.

Our key area of focus for analysis for step 3 was looking at how the pillars and priorities were correlated with Braiding and with Engagement as well. However, for completeness, we included IK methods utilized, WS methods utilized, research subject (e.g., species or ecosystem), and ecological scale of the research in the K–W tests for associations between each Braiding and Engagement. We did so to assess if these relationships could be helpful in understanding braiding practices (e.g., if studies that focused on caribou braided at more stages of the work than, say, studies that focused on polar bears).

Note also that we included all the proxy variables in Fisher's Exact test, whether they were correlated with Braiding and/or Engagement, or not. However, we only looked at associations between the proxies that were correlated with Braiding and/or Engagement, with one exception. We looked at associations between the correlated variables and year of publication because we were interested in whether the pillars and priorities correlated with the publication of the Truth and Reconciliation Commission (TRC) in 2015.

Additionally, we coded and reported on the number of case studies that discuss Indigenous values as a broad category separately from the pillars, priorities, and indicators for braiding and provide some examples of the values discussed in the literature. We report this broad ‘values’ category separately from the pillars and priorities so that we have a catch‐all code for any discussion in articles that aligned with Indigenous values (e.g., kinship with flora and fauna, water as life, core elements of Indigenous epistemologies). We felt that this was a sort of basic metric for exploring the literature and allowed us to see some important aspects of works that our more narrow coding did not.

### 2.5 Roles for IK and WS Across Project Stages

We coded the roles for IK and WS across project stages to demonstrate what braiding can look like. We then explored the coded roles for IK and WS using a combination of histograms and Sankey diagrams. Additionally, we developed pictorial icons for each of the roles for each IK and WS and how they were brought together in the literature to provide a tangible representation of the roles. This was done in collaboration with Vincent Designs Inc., an Indigenous‐led company based in Winnipeg (Table 1).

As a first step, we simply visualized what roles each IK and WS can have at each project stage through histograms. Next, we looked more in depth at what braiding could look like in studies where braiding and engagement were extensive. We pulled studies that braided IK and WS at most project stages (3/4), and where the engagement composite score was 0.5 or higher, and mapped these onto a Sankey diagram. Last, we explored whether IK and WS were used to address the same or different questions, or both, at each project stage, also through histograms.

## 3 Results

Responses to many of the questions we asked in the interviews and sharing circles answered the guiding questions for this work, *if/when and how to braid*, and we identified a suite of pillars and priorities for braiding. Participants' responses to the introductory interview questions about Indigenous and Western knowledges and what role participants thought they should have in caring for the land were foundational to understanding their responses to our more direct questions about braiding, and also unexpectedly provided methods for braiding IK and WS. Thus, we summarize some outcomes from the introductory questions together with outcomes from the direct questions about if/when and how to braid. We then braid interview/sharing circle outcomes with coded information from the systematic literature review to identify indicators for braiding. Subsequently, we briefly outline outcomes from a broader coding for whether Indigenous values were discussed in the literature. Lastly, we discuss the roles that IK and WS can have across project stages.

### 3.1 Indigenous Knowledges

#### 3.1.1 The Meaning of Indigenous Knowledges and Their Role in Caring for the Land

While many participants felt that IK, Traditional Ecological Knowledge (TEK), or an iteration of the academic terms worked fine to describe their knowledge, most participants also expressed that the word they would use in their language is ‘knowledge’ (e.g., *Anishinaabeg gikendaasowin*, word for Anishinaabe knowledge), and also ‘place‐based’ (e.g., *Eeyou Kiskeyitamowin*, the James Bay Cree word for knowledge of their particular community) (Bowles, Khan, et al. 2023). We will therefore refer to Indigenous knowledges (IK) and use the plural ‘are’ throughout this report to respect the plurality of place‐based knowledges.

IK have their own knowledge governance systems that include instructions, culture, traditional practice, and science, all working on shared principles, including relationality and responsibility (Reo et al. 2017) (Box 1). A central theme in many of the answers was the importance of lived experience, the living or enacting of that knowledge, and that IK forms a central part of Indigenous societies and cultures. Elders with decades of lived experience were mentioned as keepers and holders of this knowledge. This expression of lived knowledge and experience makes IK challenging to define as a singular and confined concept, as it is the equivalent of describing life itself, and is, at its core, an all‐encompassing way of life.

Another thread in responses to what IK are and the role that IK should have in caring for biodiversity and the environment was the responsibility for all people of a Nation to carry knowledge as a collective (Box 1). Further interpretation of this sentiment might be that relationships are inherent to IK, as is the responsibility to both humans and non‐humans.

BOX 1 **Terminology.**

**Indigenous Knowledges as Defined by Participants**

…I see it [as] Heiltsuk knowledge, or Nuxalk knowledge or Ucluelet knowledge, whatever Tribe you're talking with, has their own system and their own beliefs and their own values. It's our traditional laws or governance system that we abide by… it is basically gathering up all of our Heiltsuk values and understandings of the world, or laws that guide our way of being in this world. It upholds accountability and all the other values that I had explained in our values to our community. —Anonymous

I think it's the way that you live. If you're living in a particular way, according to a certain set of principles and values. It's often not something that you can separate out from yourself. …it has so much to do with how you live and how you take up those kinds of responsibilities that embody that knowledge. —Deborah McGregor, participant and co‐author

In Anishinabek worldview, we look at the body as a whole: the mind, the heart, the soul, and the physical, the spirit and the physical. —Sue Chiblow, participant and co‐author

Anishinabek gikendaasowin is Anishinabek people and all of our knowledge put together, because that [Anishinabek gikendaasowin] goes back to that concept that not one person can carry all of the knowledge because it would harm that person. —Sue Chiblow, participant and co‐author

*Note that while we used the term ‘Indigenous knowledges’ throughout, this term is broadly inclusive of community knowledge. For example, one community member spoke of being not very connected to their culture, but then continued to speak of hunting and fishing.

**Western Science as Defined by Participants**

Western science for me is data, it's numbers, it's compound chemicals, it's biology, it's the reports and studies, it's working within parameters. Western science is technical and it also is pretty much the only thing that people will listen to. It's the basis of all decisions. —Anonymous

When I think of Western science, I think of naming species and sub‐species and genus and labels and separation and hyper‐focus, and not necessarily looking at the larger interconnection or the larger ecosystem, and our human space within those, because they're very different than in Western science. —Anonymous

Honestly, exploitation, self‐serving, sometimes violence. I just think extraction is the main one, the main word that comes to mind. —Anonymous

Western science generally doesn't think of the earth as being alive and having agency. …They're still trying to understand why is this phenomenon happening? Science is a systematic way of trying to understand something. It has its very particular methods. Also, science to me is not homogeneous… Believe me talking to a mathematician, theoretical mathematician…. Very different than talking to a biologist, or ecologist, or wildlife person, or a person who goes out and counts birds. It's very diverse as well just like in Indigenous knowledge systems… —Deborah McGregor, participant and co‐author

**On terms for Bringing IK and WS together**

There are many words for utilizing multiple knowledge systems simultaneously, with bridging, braiding, and weaving preferred by some. Interpretations of these metaphors vary. We wish to highlight that IK and WS can be brought together at any or all stage(s) of a project and can be utilized to assess the same or different question(s), used in a complementary way or with each depending on the other. Additionally, interpretation of the terms ‘bridging, braiding, and weaving’ may depend on a person's background. For example, an Indigenous person who is a scientist and raised with both IK and WS may not have to “apply” a different knowledge system; in this case, utilizing both may be fluid. We also note that although these terms imply that IK and WS are given equal weight and brought together equitably, this is often not the case. We will show one example of the power imbalance with variation in engagement with Indigenous communities. As mentioned in the introduction, we have used ‘bridging’, ‘braiding’, and ‘weaving’ interchangeably herein.

Speakers shared spirit as an integral part of maintaining good relationships and spoke to the need to listen for these teachings. Participants shared that the connections between all beings through spirit inform how to act in the best way.

There's this connection that is more spiritual and on a different level of energy, and so in terms of reciprocity, for example, if I'm to harvest a certain plant, it's to do it in a way that won't damage those plants. —Anonymous

The medicine people, as far back as I read, try to look after two kinds of illness. One was the physical and one is spiritual, so the mental as we call it today. Once they determined that, then they would find how to treat the patient, how to treat the individual that was ill, but always bearing in mind that everything was connected, that our spiritual well‐being and our physical well‐being were connected. —Anonymous

There is no way to adequately encapsulate the role for IK in caring for the land because, as discussed above, IK are the living embodiment of that knowledge (McGregor 2004). IK are commonly expressed as action‐oriented (knowing), rather than simply holding knowledge that is not enacted. However, as an attempt to summarize what participants shared, stemming from IK having their own governance systems, IK have guiding principles through the living of that knowledge, including observing, listening, respecting.

I've never heard Indigenous people say, ‘We manage it.’ …really, we don't manage the environment. We try to contribute in a helpful way. The environment actually manages itself. It managed itself for a long time before mankind tried to manage it. We saw some of the results of mankind managing it. Not too attractive. We try to understand the environment and appreciate that for every action you take, that there's going to be a reaction. —Anonymous

#### 3.1.2 The Meaning of Western Science and Its Role in Caring for the Land

What Western science meant to speakers varied from a quantitative framework or tool used in environmental studies to an exploitative practice that affected the environment and culture negatively, while others spoke to the benefits of advanced medical science (Box 1). Others spoke to the different dimensions of IK relative to WS, such as the unknown dimension that is a part of IK but not WS, and the importance of those different dimensions. A thread through many comments was the perceived objectivity of WS and the consequences of this perception, and the reductionist nature of WS relative to IK. The context of industry and colonization (i.e., cultural genocide caused by residential schools and oppressive policies) loomed large amidst some responses, as many speakers' experiences with Western science were tied to these realities (TRC 2015). Participants shared a wide range of perspectives about Western science in a single answer, and many contrasted their ideas about WS to IK. As with IK, some participants' perspectives on WS also described the methods of WS. A less commonly expressed idea was that WS is not homogeneous.

Despite the varied feelings and perceptions expressed about WS, reflected in the following quotes, most participants saw a role for WS in caring for biodiversity and the environment, particularly as a complementary set of tools to support IK. However, many participants shared that WS needs to be guided by IK. Several participants said that WS can be used to address things like situations that Indigenous Peoples had not encountered historically, and therefore where IK has not been developed (e.g., novel environmental problems).

…yes [Western science does have a role to play] simply because there is a disconnect to what my father knew and understood to what I now know and understand. I would depend on other science studies because I need to find a way to reconnect to the earth, and as I'm doing it, there'll be some things that I don't understand, and so Western science might be helpful in making sure that things survive… —Anonymous

Sometimes, it's helpful to substitute what was lost in a messed‐up way, like what colonialism and what residential schools and that whole era took away… but I don't think that science can answer for the governance or management role. I don't think it's a substitute for our traditional knowledge of our own territory… —Anonymous

Participants expressed the importance of leading with IK, tying back to the core values, principles and laws of IK that are key to caring for the land.

I know there's a lot of benefits and there's a lot of great things that we can use Western science for, but we definitely should be prioritizing Indigenous knowledge, not only in the content but also how we design the research when it comes to science… there are studies that are done that don't kill things or don't add pieces to the animals without their permission… having the Indigenous knowledge first should be, in my opinion, the way that it evolves now… I know we're heading there. Y'all are doing this research to hopefully head that way. —Anonymous

Participants challenged the commonly held view that WS is objective while IK are subjective, expressing their view that IK are more objective and clear while interpretation of WS is subjective. This clarity in how IK are interpreted is reflected in how participants defined IK, the laws written into it, and its role in caring for the land.

You and I could interpret something completely different that's the same thing. That's the one thing that's totally different than Heiltsuk knowledge. Heiltsuk knowledge is Heiltsuk knowledge. It's there. There's not a lot of interpretations of it. It's clear, clean‐cut, but science can be interpreted so many different ways by so many different individuals that it could mean something totally different to someone else and not always a good thing. –William Housty

Other participants shared uncertainty around the success of bringing together knowledge systems and spoke of the importance of keeping the knowledge systems separate, in no small part due to negative experiences with Western science practice.

That remains up for debate [whether Western science and Indigenous knowledge systems should be braided]. … growing up I would hear a lot of Elders say not to share too much when it comes to our traditional medicines because they fear that those medicines could be exploited, because of what happened in our long history with the land. We try to protect what's still with us right now…. We try to be open, but at the same time, we want to hold our knowledge and traditions and ceremonies sacred. I believe to some extent Western knowledge can help improve things traditionally and vice versa. —Anonymous

The above quote speaks to the importance of maintaining the integrity of both knowledge systems with them interacting if and when it is beneficial, as is clearly explained in so many Indigenous‐led treaties and models (Reid et al. 2021). The quote also speaks to the importance of the “relationship” between IK holders and scientists and fear that IK will be exploited.

Unfortunately, a recurrent theme throughout the interviews was that IK is not taken seriously by decision‐makers while WS is, and that few models exist to improve the relationships between IK holders and scientists.

I touched on the weir at one point, of them building a dam to raise the water levels. [There was] science that went against what we believed in. Now we've proven them wrong and now they're starting to change things. …Yes, traditional knowledge could have been used in the past prior to development, but we haven't been asked yet. We're almost waiting to be asked because it's [development is] affecting us and affecting our way of life considerably, for our children also. —Anonymous

The need to work together and build better relationships across knowledge systems in order to solve challenges was present in almost all the interviews.

#### 3.1.3 Utilizing Indigenous Knowledges and Western Science Together

Most participants had experience bringing IK and WS together in some way, very often in response to proposed industrial development, but examples ranged from population and environmental monitoring, to industry, to education (See File S1 for a few of the examples shared). Participants' experiences using IK and WS together varied by community, Nation, and territory, with braiding occurring at various stages of research, monitoring, or management (design, data collection, analysis, reporting, and decision‐making). Participants from more than one Nation shared that both IK and WS were used in most of their projects. However, details of who was involved, about the process, or what it was like at the beginning, middle, and end were not explained in a lot of depth for any examples—aspects were touched on by some participants.

#### 3.1.4 Challenges With Bringing Indigenous Knowledges and Western Science Together

Yes, I think the difficulties that I foresee are that Western science or scientists or researchers or whoever is making these decisions or gathering this information… takes the pieces that make sense or fit into whatever their end goal already is… taking it out of context, and not having Indigenous Peoples in charge of that TEK… That's where it starts to get problematic, is when they're not actually two separate frameworks or world views. —Anonymous

Although not all speakers had experience braiding IK and WS, all were asked about experiences or foreseen challenges with doing so. While all participants spoke about the early challenges of incorporating both knowledge systems in environmental care, some communities have developed monitoring programs that have grown to use both, while others continue to have obstacles with development, government, and proponent decisions.

Participants also expressed challenges resulting from the epistemological differences between IK and WS and those differences not being respected by Westerners.

As a result of the continual impacts of colonialism and colonization our identity is often being pulled and picked out in various different ways. For Anishinabek people who are in the sciences, it's very hard to be true to who you are and take care of yourself when typically science is the opposite of that. When I think about Anishinabek knowledge, it's at the center of who we are as Anishinabek people, and when we don't have that connection of our identity and our knowledge together, that often leaves the individual, the person, in quite a tricky and sticky situation. —Anonymous

Reflected in the following quote, several participants expressed different aspects of the problems with education within broader Canadian society.

There's still really a lot of racism towards Indigenous People as being primitive, as not really knowing stuff… a lot of the programs are geared towards getting the ignorant, primitive people up to speed, so that they'll be on par with everybody else rather than trying to understand where it is that Indigenous people are coming from.

… There's still these big reports that come out like TRC, Murdered, Missing Indigenous women and girls, and others. They basically say [that] Canada is still a pretty racist colonial society. Unless you're educating people through their whole lives and career they are going to hold those attitudes. —Deborah McGregor, participant and co‐author

Discussion of power dynamics and the dismissal of IK as a challenge for braiding knowledge types paralleled the discussion surrounding the role for WS in caring for the land.

I worked on the Great Lakes files for a long time, very difficult to have traditional knowledge there. People would go, ‘we've got the state of the Great Lakes conferences’, and that's where you're trying to assess the health of the Great Lakes. Indigenous knowledge was so hard to even get to the table. Then, it would just be completely marginalized…none of it would ever show up in the report. Even with change, now with the re‐negotiation of the Great Lakes Water Quality Agreement…. Maybe now you get a little box on page 103. —Deborah McGregor, participant and co‐author

Even for fish species. The traditional knowledge of where the sturgeon are? Why are they there? We know why they're there, but in order for us to tell the MNRF [Ministry of Natural Resources and Fisheries] …you got to do those experiments and studies to show them the things you already know. —Anonymous

Participants expressed that IK was ‘used’ to fulfill a proponent's mandate rather than for community purposes, a demonstration both of the dominance of WS, and the extractive nature of industry. The role of WS in the following quote is not stated, but we include the quotes with the assumption that the WS studies form the foundation of the assessments done.

Lots of traditional land use studies when industries go in and need to get project approval. That's pretty much the driving factor, it's never, ‘Let's have traditional land use studies because there's still people using the land traditionally.’ —Anonymous

Problems with outsiders' process in working with communities continue as well, with inadequate understanding of when consultation is needed, or what meaningful consultation is.

Additional ongoing systemic barriers discussed included political decisions and laws making traditional ceremonial practices illegal and funding/timeline pressures.

One of the big things in Alberta that we have issues with is, if we're doing a cultural camp or sharing this knowledge, …if we set a net we can only share with our family, your immediate family… How do you show somebody how to fillet a fish if you can't share it with your community members? If a fish and wildlife club were to come out, you'd be fine. People distrust the system already… It's always on the hush hush when there's cultural camps. How do you educate people with skills and skill development and skill practice if it's illegal? —Anonymous

Other challenges discussed included the historical context of maltreatment leading to community‐wide hesitation.

I think the only difficulties going forward [are] going to be those barriers and those fears of engaging with each other. We've got to break those down and start collaborative engagement, reaching out to First Nations with institutions and academia. —Anonymous

In summary, our thematic coding showed that challenges with braiding included:

1. IK not being trusted or taken seriously by non‐Indigenous Peoples or systems;
2. IK being extracted from communities by external professionals (e.g., researchers and colonial governments) and not treated with respect;
3. Capacity issues within communities to engage with work being proposed by external professionals;
4. Challenges with political will of colonial governments to allow cultural activities; and
5. Mistrust of communities toward external professions due to maltreatment in past and present

#### 3.1.5 Overcoming Challenges with Bringing Indigenous Knowledges and Western Science Together

Most interviewees suggested solutions, or ways to address challenges with braiding knowledge systems, with the prevailing theme being a commitment to long‐term relationship‐building. We outline many solutions here (e.g., partnering early, connecting often, listening, returning the knowledge that was shared), and also emphasize that IK and key values therein should lead (i.e., guide) research and monitoring work. Some of the values that were discussed in relation to caring for the land included reciprocity, openness, kindness, listening, and challenging our preconceived notions. Education about how to be respectful and go about collaborative research was also emphasized.

Building and maintaining strong relationships was the most common solution to challenges with braiding presented by participants.

A lot of it is relationship‐building…, because trust is a huge thing. They [Indigenous Peoples] distrust the system, so how do you share this knowledge? They [communities] think you're going to take it [IK] and run with it and exploit it… —Anonymous

Some stakeholders and groups of these societies are scared to even engage or interact with each other. I see this and I think we need to remove those barriers and really remove these preconceived tensions perhaps that don't exist. Then that meaningful engagement and working together with projects and basically creating case studies for your region, I think that's the excellent way of doing it. Sitting down and looking over each other's values and needs, and essentially [asking] what are your asks, and how are you going to amend that to, and to realize that there's no other option but to move forward really. —Anonymous

There was a really interesting paper done by Teresa Ryan for the AFN [Assembly of First Nations]. …She had a good point, she goes, ‘People keep saying knowledges are interacting, Indigenous knowledge and Western knowledge, but it's not. It's people who are.’ —Deborah McGregor, participant and co‐author

Part of being in relationships is understanding in what context and for what purpose knowledge has been given to you.

Yes, and maintaining those relationships. Like, if I were to tell you about a specific plant and its medicinal purposes, it doesn't mean that you've been given the authority to pick that plant and use it. —Sue Chiblow, participant and co‐author

We need to bring the two worldviews together. We don't necessarily need to integrate them with one another, but like the Two Row Wampum Belt, we're both traveling in our own journeys but intersecting and then coming together when we need to do environmental problem‐solving. That would help alleviate a lot of conflict that's happening. When there's a true, respectful, meaningful relationship we can work together to address species at risk, biodiversity, and all those types of things. People need to be open‐minded and have that respect that our knowledge is just as valuable as Western Science. —Sue Chiblow, participant and co‐author

Engaging early and consistently was also a priority.

I think really the best way of doing it is just trying to have as much community input as possible, versus just doing a couple interviews here or there. It's just, if you almost have the community input at each stage of the project, then you get more and more of that traditional knowledge into the project. —Anonymous

Taking the time and being present consistently are needed to build trust.

For example, we have teachers, nurses, and doctors who practically technically live here to be with our people. The teachers are with our youth, the doctors and nurses are treating basically everybody. Ones that do take an interest, that invest their time living here, even they created that trust with the people that they've learned some things that is a privilege to know. That took a number of years for them to reach to that point. …Some get invited to some of our gatherings or just to be in the bush with them, just to have a little fire and food. —Anonymous

Education about history, roles and responsibilities will help outsiders understand context for work better, and to have an understanding of the culture and knowledge they are working with. Work on the part of outside institutions as well as Indigenous Nations is important.

…I think there really needs to be a lot more education at the institutional level and at the individual level so that everybody's aware of the processes that exist [in the community for conducting research]. We have started to do that a little bit here through protocol agreements with them [students]… those sorts of things are important at that larger institutional level [so] that they [outsiders] understand what's being done and that they're not just signing off for individuals to go run around and do whatever they want but there is actually guidelines that are in place to follow. There's even a big role for Heiltsuk to play …finding a way to educate people about processes. —William Housty

…I think consent is definitely another big part of that relationship building. …who you're asking consent for when you're doing research… it can be very difficult when some community members are open to it and other ones aren't, or those voices aren't necessarily reflected in Chief and Council, for example, or the land office employees.,…having that relationship building allows researchers and communities to have those kinds of conversations… but if a lot of community members are interested in it and agree with the researchers, then maybe that can sway Chief and Council. —Anonymous

In relation to people within an Indigenous community, not with Western scientists per se, one participant said the following.

My honest feelings, when I said the truth hurts, is that we have to maybe change the way we think about each other. We need to protect the planet together, and we must share our knowledge to protect the planet together. Maybe she's [a female Elder] right when she said we are part of that destructive force. We need to change the way we think, the way we do things. We need to trust each other. We need to work together. We need to listen together. —Anonymous

As discussed above in the Indigenous knowledges section, to build good relationships, the representation of women, youth, and Elders in any knowledge generating project is important. Knowledge is meant to be shared and held by entire communities, with different community members holding different responsibilities (Craft 2018; McGregor 2009, 2014, 2021). Thus, as many community members as possible must have representation in the curation and direction of it, according to a communities' wishes.

I think when we talk about the *Anishinabek gikendaasowin* [Anishinabek people and all their knowledge put together], ensuring I suppose that we also speak and try and keep a balanced approach, that we're also speaking with women. Because women have a different set of knowledge because of their roles and our responsibilities. …It's the typical, and you look at the history books, and it's always the Jesuits or the explorers who are male always talking to the males. —Sue Chiblow, participant and co‐author

Again, going back to our young people, I see that they're anxious to get their boots on the ground and get moving and start educating larger society and our own community and our own people on many things, including Indigenous priorities and the priority of managing or helping the environment. —Anonymous

If you had an Elder interviewing an Elder in Cree, that would be the ultimate knowledge transfer. —Anonymous

An important aspect of equitable representation is intergenerational knowledge transfer. Many speakers shared the importance of helping communities build resiliency through facilitating spaces where Elders and youth can gather to share knowledge.

Anytime he [the Elder] wants to do any land‐based work, he takes the youth out with him because no matter what he's doing, he's always teaching them about everything on the land. He's on the land, he's an open encyclopedia, right? —Anonymous

And the following quote encapsulates the importance of community‐led work and language revitalization as critical to developing capacity within communities to engage in this work.

Colonization has been so devastating in communities, and so support communities to revitalize it [their knowledge], like language programs. Like I said, I'm on this Adjudication Committee. The thing is the ones who get the funding are the ones who conform to the government agenda. The ones who really want to do their own thing, they're not supported. …[communities] need to be supported appropriately, in the way that they see fit. They [funders] have to start looking at language revitalization as being critical to conservation, but they don't. Like I say, language revitalization is critical to climate change because it builds resilience in community people and young people. To me, that's what needs to be done because right now, it's not equal and it's not on par. —Deborah McGregor, participant and co‐author

We distilled the solutions presented for overcoming the challenges with braiding knowledge systems above in the quotes to the following pillars and priorities:

1. Build and foster relationships: Cultivating relationships requires good listening and learning from past mistakes, as well as allowing time for development of trust.
2. IK should guide projects: WS has a place, but projects need to be guided by IK.
3. Indigenous communities should lead projects: Outsiders (researchers, industry, NGOs) need to support communities to lead the work according to what communities determine is needed.
4. IK must be respected equally with WS: IK is based on experience, observation, and replication over generations; the integrity of both knowledge systems must be upheld, and IK needs to be given the same weight as WS.
5. Embrace reciprocity to the land and one another (focus on people): There needs to be give and take between IK holders and Western scientists, for example respecting who has knowledge in a particular context. Give and take also means outside practitioners need to put work into understanding as many of the multi‐dimensions of IK as they can, and give back that knowledge to the land. Additionally, outside practitioners need to return knowledge to communities and demonstrate doing something good with the knowledge that was shared.
6. Embrace responsibility to the land and one another (focus on land): Braiding work to respect the land and each other (i.e., not taking too much) to ensure continued access to the land.
7. Ensure equal gender and age representation (i.e., youth and elders): As many genders and age groups need to be included in braiding work.
8. Intergenerational knowledge transfer is important: Explicitly involve teaching between generations in braiding work.
9. Language revitalization is critical: Revitalization needs to be prioritized in braiding work because IK is held within the language.

Perhaps predictably, these pillars and priorities are not independent from each other, or from foundational principles and values for caring for the land (Menzies, Bowles, Khan, et al. 2022)—rather, they exist in relation to one another. For example, reciprocity was often coded for the same text as gender and age representation. We separated some of the meanings in the above list, but ultimately they are all in relationship with one another. The importance of strong relationships was the most prevalent theme throughout all of the knowledge systems and braiding questions. The quotes we have shared in this report on how outsiders can help and what role Western science can have speak to the need for IK and the value systems therein to guide research, monitoring, and management—this theme speaks inherently to the need for this work.

### 3.2 Braiding the Knowledge Shared by Interview Participants With Coded Literature

#### 3.2.1 Indicators for Braiding

**FIGURE 3:**
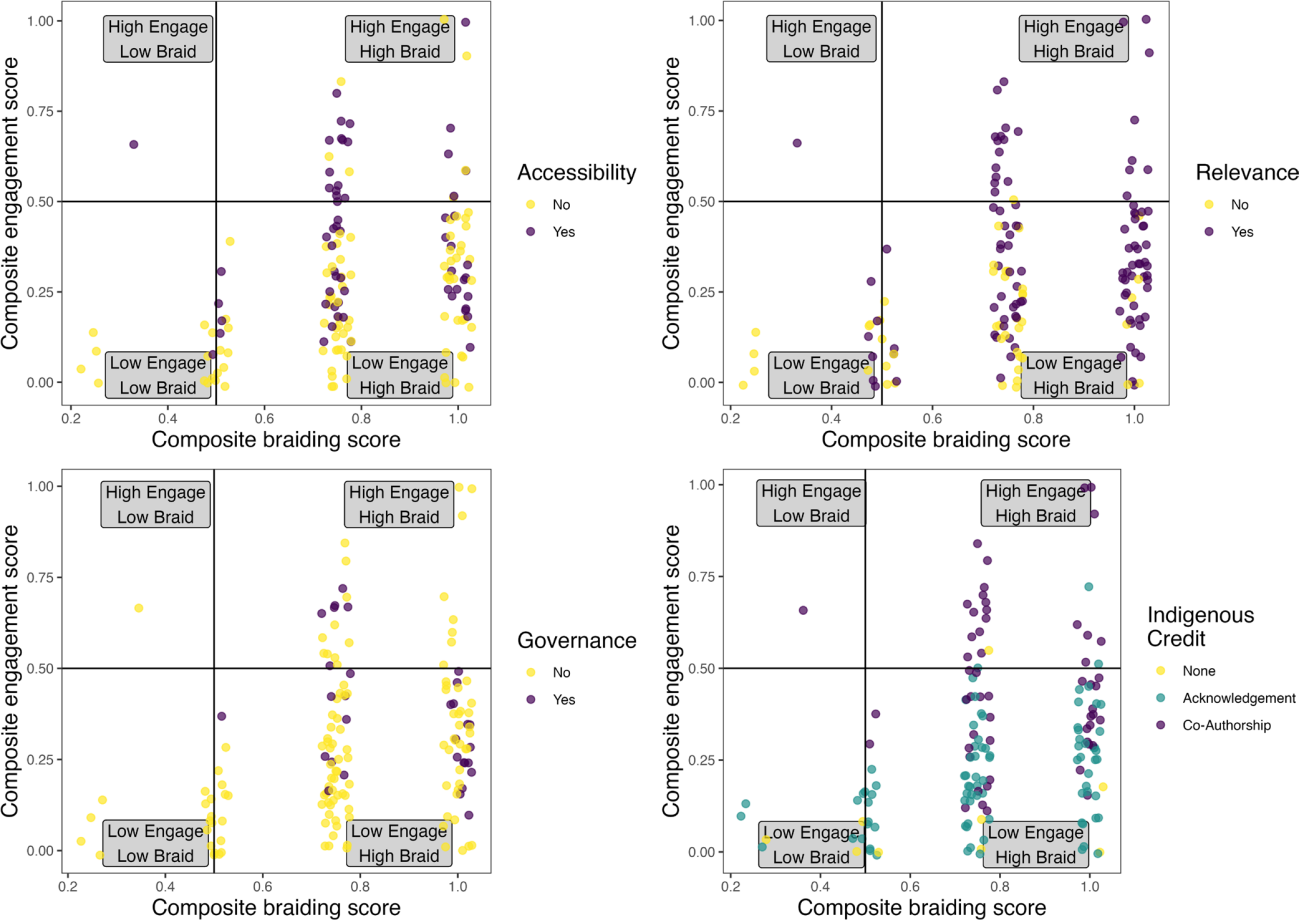
Distribution of pillar and priority indicators with the extent of Braiding IK and WS and Engagement with Indigenous communities across project stages, across all coded studies (*n* = 150). The indicators are the variables identified as the best proxies for each of the pillars and priorities that the interview/sharing circle participants shared with us. Composite Braiding scores on the x axis and composite Engagement scores along the y axis represent the scaled composite score across project stages (design, data collection, analysis, and reporting and decision‐making) for each study (each study is one dot). Indicators are: Engagement, or to what level do Indigenous community members have authority in each stage of a project; Accessibility, or are findings accessible to Indigenous community members (e.g., findings shared in community, data available to/stored with community members, local publications produced, disseminated in local language); Relevance, or are findings reported in the context of concerns, issues or interests defined by Indigenous community members; Governance, or does the study address concerns related to data sovereignty or information governance. See Table S3 for examples of studies from each of the quadrants in the figure.

The extent of engagement between study authors and Indigenous communities (Engagement) was the best proxy for the first three pillars and priorities: projects need to be community‐led; WS has a place, but IK needs to guide projects; and IK must be respected equally with WS (Table 2). We found that the composite scores for each the number of stages at which braiding occurred across projects (Braiding) and the level of engagement with Indigenous communities across the stages of a project (Engagement) were correlated (Kruskal‐Wallis; *p* = 0.007) (Table 2, Figure S1). Overall, looking at the distribution of studies in the braiding space, most studies utilized both IK and WS at most project stages (Figure 3). Additionally, high degrees of community engagement almost always indicated braiding at most project stages (Figure 3).

**TABLE 2:** Pillars and priorities for braiding IK and WS as identified by participants (left), the questions that we coded in the literature that best represent each pillar and priority, the proxy variables we used to represent those coded questions, whether each proxy variable correlated with composite scores for Braiding (B) or Engagement (E), and then how proxy variables that correlated with each braiding and engagement correlated with Indicator variables (Relevance (R), Governance (G), Accessibility (A)). Where a single proxy variable is given for multiple pillars and priorities, that proxy variable best represents all of those pillars and priorities. Where multiple proxy variables are listed for a pillar and priority, each proxy variable was treated independently. Proxy variables that are indicators for braiding are in orange.

Pillars and priorities	Coded questions that best represent pillars and priorities	Proxy variables for the coded questions (with the indicator variables in colour)	Proxy variable correlation with braiding (B) and/or engagement (E)	Indicator variable correlations with other proxy variables
Indigenous communities to lead projects	Are Indigenous community members included in the decision to initiate the study?	**Engagement** (a composite score of responses to the six coding questions)	B, E	R, G, A
IK should guide projects	To what level do Indigenous community members have authority in each of the following, where each was ranked from 0‐4:			
IK must be respected equally with WS	In setting project objectives and research questions? In the research design? Regarding the implementation of the research? Regarding the analysis of the research (data analysis, interpretation, evaluation)? Regarding the dissemination and/or application of the research?			
Build and foster relationships	Is the study built on a previous relationship or collaboration with an Indigenous community?	**Previous_relationship**	E	R
Ensure equal gender and age representation	Demographics of knowledge holders—elders	**Demographics_elders**	E	G
	Demographics of knowledge holders—Age	**Demographics_age**		
	Demographics of knowledge holders—gender	**Demographics_gender**		
Intergenerational knowledge transfer is important	Within themes identified in each manuscript, those that were related to intergenerational knowledge transfer or revitalization of Indigenous knowledge	**Intergen_knowl**		
Embrace reciprocity to the land and one another (focus on people)	Are findings accessible to Indigenous community members?	**Accessibility**	E	G
	Are findings reported in the context of concerns, issues or interests defined by Indigenous community members?	**Relevance**	B, E	G
	How were Indigenous community members credited for their knowledge contributions and efforts?	**Indigenous_credit**	E	R, A
	Do authors acknowledge following community protocols?	**Community_protocol**	E	R, A
	Do authors acknowledge that participant consent was sought?	**Participant_consent**		
	Do authors acknowledge that community consent or review was sought?	**Community_consent**	E	G, A
	Is the study building on a previous relationship or collaboration with an Indigenous community?	**Previous_relationship**	E	R
	Does the study address intellectual property rights or risks for Indigenous communities?	**Intellectual_property**		
	Does the study address concerns related to data sovereignty or information governance?	**Governance**	B, E	R, G, A
Embrace responsibility to the land and one another (focus on land)	Within themes identified in each manuscript, those related to ensuring continued access to culturally important species	**Cultur_import_spp**	B, E	R
	Within themes identified in each manuscript, those related to enacting IP governance and responsibilities or territorial protection	**Intellect_prop govern_&_territ_protec**	E	
Language revitalization is critical	Language was not coded	None	NA	NA

Addressing the other five testable pillars and priorities (Relationships, Gender and age representation, Intergenerational knowledge transfer, Reciprocity, Responsibility), at least one proxy variable that we identified for each pillar and priority was correlated with Engagement and/or Braiding, except for Intergenerational knowledge transfer. Of the 20 proxy variables tested, ten proxy variables were correlated with the composite score for Engagement, while three were correlated with Braiding (Kruskal‐Wallis tests: Table 2, Figure S1). Relevance (are findings reported in the context of concerns, issues or interests defined by Indigenous community members) and Governance (does the study address concerns related to data sovereignty or information governance) were correlated with both Braiding and Engagement.

Fisher's Exact tests showed that many of the proxy variables that were correlated with Braiding and/or Engagement were correlated with one another (Figure S2, Table 2). Thus, we made groups of variables based on the overlap of significance. Visualizations of each variable that was correlated with Engagement and/or Braiding reflected that from each grouping of variables, Engagement, Relevance, Governance, and Accessibility showed the associations with Braiding and Engagement most clearly (Figure 3). It is these four variables that we identify as the indicators for Braiding. When Engagement, Relevance, Governance, and Accessibility are taken into consideration in carrying out a project, studies generally engage and braid at most stages of the work and reflect the majority of the pillars and priorities for braiding (Figure 3). We have added a visualization of Indigenous credit in Figure 3 even though it is correlated with Relevance and thus in the Relevance group of proxy variables, because the patterns of association with Braiding and Engagement are so clear. We also note here that while Intergenerational knowledge transfer was not correlated with Braiding or Engagement, very few studies discussed it; with more data points, Intergenerational knowledge transfer may be correlated.

Interestingly, WS methods were correlated with the extent of Engagement, but none of the remaining additional variables that we tested were correlated with either Braiding or Engagement (i.e., year published, research subject, ecological scale, methods for IK collation, and methods for WS data collection) (Figures S1 and S2).

The above discussion focused on the values associated with the pillars and priorities; however, many other Indigenous values were addressed in one way or another in the literature. Indigenous values as a broad category were discussed in 103/150 case studies, and values varied widely. For example, a “yes” code for discussion of values could range from discussion of Indigenous food systems to discussion of Indigenous worldviews. Clearly reflected in the quotes shared above, much of the discussion with interview participants centered on values (Menzies et al. 2024).

### 3.3 Roles for Indigenous Knowledges and Western Sciences Across Project Stages and Methods for Collation of IK and WS Data Collection

**FIGURE 4:**
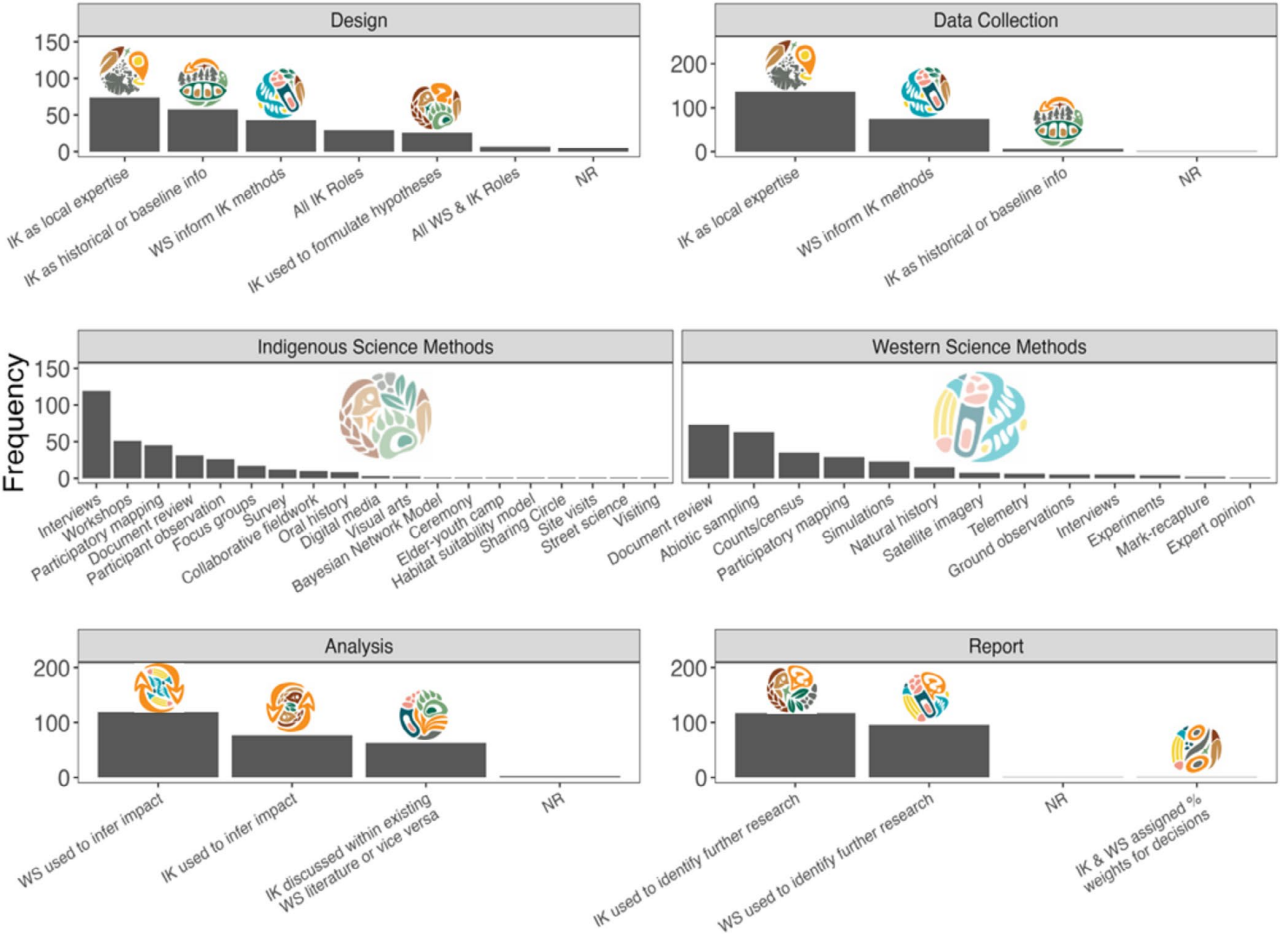
The roles for each IK and WS at each project stage (upper two and lower two plots) and methods utilized for collation of IK and WS data collection (middle two plots) across all 150 studies. A single study could have multiple different roles and thus be accounted for in multiple categories within a project stage. Note the different scales on the y‐axis. Different y‐axis scales allowed the visual icons to be a consistent and legible size. The roles shown are what each knowledge type was utilized for. The abbreviated roles include the following: use IK as local scale expertise; IK as a source of historical/baseline information; IK used in formulating research questions and hypotheses; WS informing IK methods; IK used in inferring impacts/changes (i.e., mechanisms‐ for example for wildlife population change); WS used in inferring impacts/changes (i.e., mechanisms‐ for example for wildlife population change); IK is discussed within the existing WS literature or vice versa; IK supporting identification of further research questions and/or management recommendations; WS supporting identification of further research questions and/or management recommendations; and IK and WS are assigned percentage weights for decision‐making. The methods plots in the middle indicate what tools were utilized to collate or collect that knowledge type (IK or WS), rather than the method being rooted in a particular philosophy (i.e., Indigenous versus Western methodology). For example, interviews utilized to collate IK were categorized as an Indigenous knowledge method even though they are a Western social science tool.

IK and WS can have a plethora of different roles across the stages of a project or study (design, data collection, analysis, reporting, and decision‐making); there is no one way to utilize them (Figure 4, Figure S3, Table S4). Across all the studies in our systematic review, at project design the most common roles were IK as local scale expertise and IK as a source of historical/baseline information. At data collection, the most common roles were to use IK as local scale expertise, and then WS informs IK method. At analysis, the most common roles were WS used for inferring impacts/changes, followed by IK used for inferring impacts/changes. At reporting and decision‐making, the most common roles were IK supporting identification of further research questions and/or management recommendations, followed by WS supporting identification of further research questions and/or management recommendations. There was a wide range of information gathering and data collection methods used at data collection. The most common IK method utilized was interviews, while for WS it was document review (Figure 4). Examples of what each of the codes that we describe here can look like in practice, with excerpts from the literature, can be found in Table S4.

**FIGURE 5:**
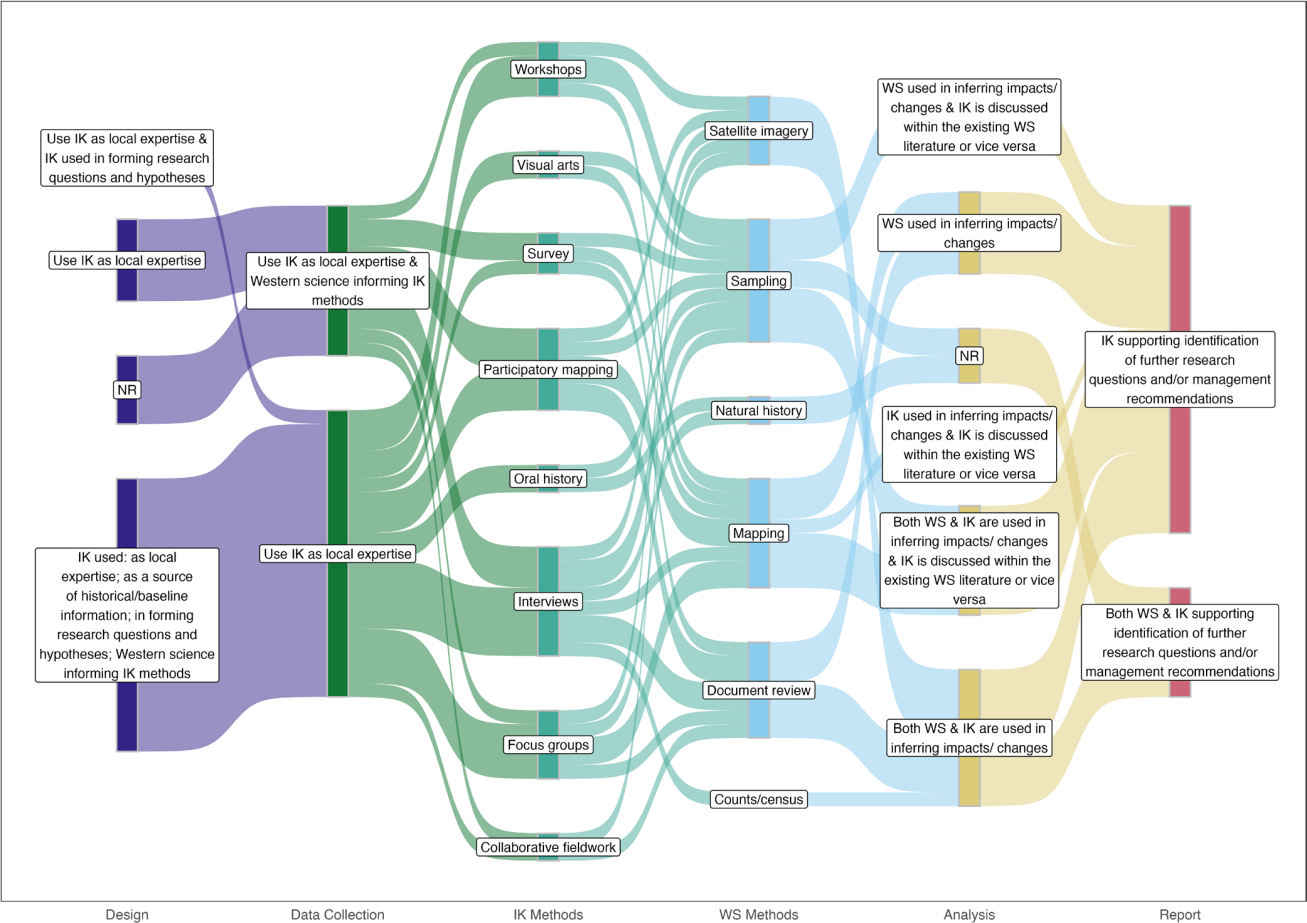
Roles for each IK and WS across project stages for the *n* = 8 studies that coded positively for relevance and governance, and that have a braiding score of ≥ 0.75 (braid at ¾ stages), with a 0.5 engagement score as well. We included methods used for IK collation (Methods_IK) and for WS data collection (Methods_WS) here as well. Each study is represented by a pathway between each node. NR (not reported).

**FIGURE 6:**
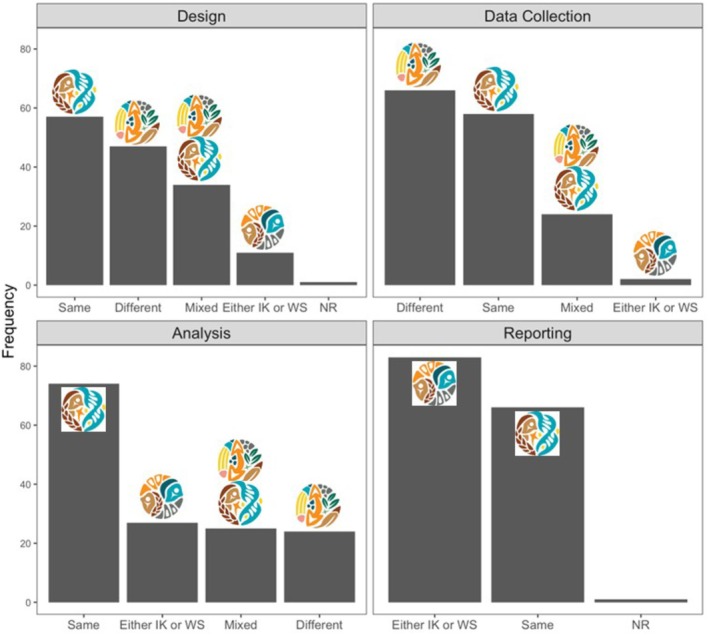
Frequency of studies (*n* = 150) where IK and WS were used to ask or do the same thing, different things, both the same and different things (mixed), or where only IK and WS were used (either IK or WS, i.e., braiding did not occur) or were not reported (NR) across the stages of a project.

To explore in greater detail what roles IK and WS can have and methods for IK and WS information collection could look like across project stages, we visualized studies that coded positively for Relevance and Governance, and that had a Braiding score of ≥ 0.75 (braid at ¾ of project stages), with 0.5 Engagement score as well (Figure 5). Relevance is the proxy variable for the question “Are findings reported in the context of concerns, issues or interests defined by Indigenous community members”, and Governance is “Does the study address concerns related to data sovereignty or information governance”. We chose studies that correlated positively for these indicators because they are the two indicators that correlated with both Braiding and Engagement and that correlated with proxy variables that represented most pillars and priorities. The eight studies that met these criteria could be considered exemplar studies for Braiding and Engagement. Braiding and Engagement were extensive in these eight studies, and there were often many roles for each IK and WS at each stage of a project (Figure 5); indeed, most of the roles and methods for IK and WS that were seen across all 150 case studies reviewed (Figure 4) were represented in this subset of studies. Also though, reflected in Figures 5 and 6 and Figure S3 there is clearly no one path for a given project.

IK and WS can be utilized to address the same questions (e.g., at data collection, what is the distribution of caribou; at analysis, how many morphotypes are there) or different things (e.g., at analysis and decision‐making, utilizing WS statistical methods for analysis and IK to guide decisions) (Figure 6). In Figure 6, “Mixed” refers to IK and WS being used for both the same and different things, whereas “different” reflects that while IK and WS were both used, it was to ask different questions or for different things.

## 4 Discussion

Overall, our dialog and exploration of themes relating to if/when and how to braid IK and Western science for biodiversity work show that there is considerable desire to do so by Indigenous Peoples, researchers, and governments at all levels in Canada. Unsurprisingly, there is no prescribed way to braid—it can be done at any stage of a project. Rather, we show that braiding must be guided by Indigenous Peoples and knowledges and strong relationships. Through conversations with Indigenous Peoples, we identified nine pillars and priorities for braiding (Table 2). In our coding of the case studies, we identified proxy variables for the pillars and priorities (with the exception of language, which we did not code because we felt it required nuanced coding and attention beyond what we could do here). We found that there are four indicators (Engagement, Relevance, Accessibility, and Governance, Table 2, Figure 3) that, when they are present and discussed in the case studies, the majority of the other pillars and priorities for braiding are also present in the work. The roles for each IK and WS that we found in the reviewed case studies may reflect both positive narratives about how IK and WS can be used and also colonial legacy, where IK is clearly not being respected as it should be. Including the interviews, analysis of case studies, and roles for IK and WS in the same article has allowed us to interact dynamically between all of these forms of information, collectively producing a more holistic analysis and toolkit for project leads.

One of the challenges with writing generally about the practice of braiding in research, monitoring, management, and policy development is considering the transferability of practices. For example, for a regional impact assessment or managing transboundary/migratory species, is it possible to extrapolate braiding practices between different communities, cultural contexts, regions, institutions, or levels of government? Or even, do the particular research subjects such as species or ecosystems matter in how we do so? One way we thought about how we could extrapolate our observations is to consider what pillars and priorities or other elements coded in the case studies (e.g., species studied, ecosystem) correlated with Braiding across all the studies. We found that neither Research subject (i.e., species, ecosystem), Ecological scale, nor Methods for IK collation correlated with Braiding or Engagement, but rather that proxy variables that were associated with almost all of the pillars and priorities did correlate with Braiding and/or Engagement (Table 2 and Figure S1); curiously, WS methods correlated with engagement as well. Another approach to thinking about the transferability of practices is through scale, which we discuss toward the end of this section.

Amongst all the findings herein, the importance of strong relationships and all that stems from strong relationships is perhaps the most important. We saw that Engagement correlated with many more pillar and priority proxy variables than Braiding. And in case studies that were relevant to community needs, authors engaged with the community and followed community interest and way of life from the research question to the way the study was conducted and results disseminated, and braided knowledges at most stages of the project. As McGregor (2021) points out, there is no one ‘way(s)’ to do braiding; relationships with communities and community‐led work are likely the only path forward because it is going to depend on the knowledge that people have of the land. IK is inherently place‐based as it is a way of living. And as reflected in the other pillars and priorities, relationships need to be founded on responsibility and reciprocity, both with other people and non‐human relations (Chiblow et al. 2024; Menzies et al. 2024). There is a swath of Indigenous scholarship discussing this fundamental value or series of inter‐rated values, including thorough and multi‐dimensional discussion on pages 19 and 20 of “The state of the world's Indigenous Peoples” (Chiblow et al. 2024), Cajete's work on natural laws of interdependence (Cajete 2000), Starblanket's work on a relational paradigm (p175–207) (Starblanket and Stark 2018), and Mitchell (2011). When a relationship is strong and there is trust, communities and outside collaborators can communicate about when and if IK and WS are important in any given aspect of the work; as we see, braiding can be done at any stage of a project (Figure 4).

Everyone interviewed shared the perspective that braiding projects (which included all or any of research, monitoring, and management) must be led with IK. There are perhaps a couple of things that can help us understand why. As we heard, there is no way to adequately encapsulate the role of IK in caring for the land because IK are the living of that knowledge (McGregor 2004); this necessarily leads to a different motivation for caring for all our relations. Additionally, peoples' knowledges are place‐based and differ across Nations, geographies, and territories (Battiste 2002; Berkes 2012; Housty et al. 2014). That work needs to be led with IK has been emphasized broadly in published literature within Canada (McGregor 2004; McGregor et al. 2020; *M’sɨt* *No'kmaq* et al. 2021), and internationally (Chiblow et al. 2024; David‐Chavez and Gavin 2018). For example, in Canada, the Yukon First Nation Elders' recognition of water as a ‘relative, teacher, medicine, and healer’ (Wilson and Inkster 2018), or the Cree recognition of the land as ‘a teacher of law and governance to whom we are accountable’ (Daigle 2016). Within this Cree concept of the land, self‐determination is constructed through kinship and ceremony rather than Western individuality. As discussed by Robin Wall‐Kimmerer, WS is very good at addressing questions of yes or no, while IK should be used to guide the work and our decisions, because IK were designed to do just that (Kimmerer 2013).

Our broader yes/no values coding elucidated the extent of discussion about values in the case studies, and the breadth of how they were represented. Values were discussed in the majority of case studies, and in very diverse ways. One example of how they were discussed is: “In contrast, our interest is in how to gauge water vulnerability with Indigenous communities in culturally specific and place‐based ways. Assessing water vulnerability with Indigenous communities necessitates a particular perspective as ‘water is the lifeblood of the land and of the Indigenous peoples and cultures that rely upon it and its waters’” (Plummer et al. 2013).

Many themes that emerged from our interviews regarding what makes braiding knowledge systems successful are mirrored in Reo et al. (2017) in the factors that facilitate successful involvement of Indigenous communities in multi‐part environmental governance. These factors from Reo et al. (2017) included “respect for Indigenous knowledges, control of knowledge mobilization, intergenerational involvement, self‐determination, continuous cross‐cultural education and early involvement”. Regarding research collaborations between scientists and Indigenous communities, Reo et al. (2017) discuss the importance of long‐term personal relationships, trust, and respectful relationships as key insights. Considerable overlap in themes was also reflected in *M'sit* *No'kmaq* et al. (2021), who identified seven factors that are key for transforming biodiversity in Canada and beyond: “…[E]mbracing Indigenous world‐views of ecologies and *M'sit No'kmaq*, learning from Indigenous languages of the land, Natural laws and *Netukulimk*, correct relationships, total reflection and truth, *Etuaptmumk* – ‘two‐eyed seeing,’ and ‘strong like two people’, and ‘story‐telling/story listening’”. Readers can look to Esquible et al. (2024) for a review and Bulmer et al. (2024) for a practical case study of international fisheries literature that echoes overlapping themes, and Mercier and Jackson (2023) for a study highlighting principles needed to better equip scientists to make space for and include mātauranga Māori (Māori knowledge).

Of note, almost all studies that had engagement scores of 0.5 or above braided IK and WS at ¾ or all stages of projects (i.e., at design, data collection, analysis and/or results and decision‐making), suggesting that more engagement leads to more braiding (Figure 3). A score of 0.5 reflected collaborative work, where collaborators and researchers work together, but with researchers having primary authority and making decisions about/facilitating the process for collaboration (Table S2). We would be remiss though not to point to the flip side of this statistic. That is, of the 150 case studies that were coded < 25 reflected engagement between the authors and the communities involved that was 0.5 or above (Figure 3) even though the majority of studies braided at ¾ or all project stages. This statistic may reflect an extractive paradigm, which is consistent with many interview participants’ experiences and is well documented elsewhere. Interestingly, year published did not correlate with engagement, suggesting that studies have not necessarily done research in a better way over time.

Speaking to the roles for each IK and WS across project stages in the case studies, clearly there can be many roles at all stages (Figure 4, Figure S3, and Table S4), which is also reflected in interview participants' focus on process rather than the exact roles that each IK and WS could take. Tying back to the place‐based nature of IK, perhaps this is reflected in the roles, with IK as local scale expertise in most studies in both the design and data collection stages. Reflecting the long‐term nature of IK, IK as local expertise was followed closely by IK as historical or baseline information at the design stage. Possibly also reflective of the dogma surrounding the relevancy of IK and WS though, WS held the dominant role at the analysis stage. It was a surprise to see that at the reporting and decision‐making stage, IK was utilized to identify further research in the greatest number of studies, though there was not a huge discrepancy between IK and WS leading this category. Our decision to code for roles in this work was largely to help provide a catalog of how IK and WS can be brought together; this catalog is in Table S4. Regarding other helpful resources for roles, Riedlinger and Berkes (2001), an international review, is what we looked to initially when developing our codes for roles (e.g., role of IK in hypothesis generation). Additionally, Stern and Humphries (2022) is a very helpful systematic review of weaving IK and WS in quantitative analyses for studies on wildlife across the globe—their analysis looks across project stages and addresses many important considerations for ‘how to’ braid for quantitative studies.

It is striking that for three of the four stages of work both IK and WS are utilized considerably more than only one or the other (i.e., the ‘either IK or WS’ category had low frequency for three of the four stages of work, Figure 6). At reporting and decision‐making it was more common for only IK or WS to be utilized. Contrary to our expectations though, IK used to generate future research questions and/or management recommendations was more common than WS (Figure 5) (but see Riedlinger and Berkes (2001) to see that this has been common for a long time). More nuanced analysis has been done on smaller subsets of papers looking at what roles IK and WS can play, and if the two can provide the same information (e.g., at the negotiation stage of Tengö et al. 2017). Results of IK and WS can be congruent (say the same thing, e.g., a given species is declining), complementary (different things that contribute to a full picture, e.g., genetic analysis of population structure of wildlife and human culture groups co‐occur), or different (reflect different outcomes, e.g., IK indicates population stability, whereas WS indicates population decline). WS often extends over larger spatial scales, while IK is more local, but extends detailed information over longer time‐scales. Additionally, IK can often provide much more holistic information about an ecosystem, whereas WS can investigate mechanisms that could explain ecosystem changes that IK cannot “see” (Bowles et al. 2022; Gagnon and Berteaux 2009; Polfus et al. 2014; Riedlinger and Berkes 2001). Stern and Humphries (2022) provide helpful guidance on addressing scale questions for quantitative analysis at an international scale. Looking at spatial and temporal scales is an important question to consider at a regional scale. Ultimately though, the pillars and priorities shared by participants are what address the question about scalability best. Focussing on the indicators for these pillars and priorities (Engagement, Relevance, Governance, and Accessibility) should help mitigate the impacts of scalability.

### 4.1 Persistent Challenges With Moving Forward in a Good Way

The theme in participants' responses that IK is not taken seriously or is dismissed by the establishment is clearly reflected in the dominance of Western science for research, monitoring, and management by academia, industry, and governments alike (Artelle et al. 2021; Hessami et al. 2021; McGregor 2018; Reid et al. 2024). Although it is not the case that all studies or work directed by Western researchers would be disrespectful, colonial legacy has resulted in the dispossession of land, as well as the suppression and dismissal of Indigenous Peoples' cultural practices that sustained vibrant ecosystems until the time of colonization (Joseph 2018; McGregor et al. 2020). There is a historical and ongoing colonial legacy that scientists and external researchers must reconcile (Wong et al. 2020). It is important for Western researchers to acknowledge this history and the context they bring with them when entering into partnerships with Indigenous communities, no matter how respectful they are as individuals.

The quotes shared about epistemological differences between IK and WS reflect a disconnect between Indigenous and Western approaches, and part of what has created distrust of working with people from outside communities. Education was raised as a challenge and concern by participants, and providing more of it can hopefully help non‐Indigenous people understand and respect IK better. The problems that participants identified with education are unsurprising, as while 36% of Canadians aged 25–64 hold a university credential (Colleges and Institutes Canada 2024), fewer of those take Indigenous studies courses. As part of the TRC's 94 calls to action, many school districts are developing educational programming, but anecdotal evidence shows that there is considerable growth needed with educational material that is being produced and delivered. To be taken seriously means that Indigenous Peoples and knowledges need to be a part of work from the beginning, and all elements of their knowledge must be used in the way they see appropriate.

It may be worth saying that Indigenous communities are not generally as consumed with “weaving” as the scientists because Indigenous Peoples already do it. It is not a conscious thing anymore, although at one time it was. Braiding/weaving becomes an “issue”‐ for lack of a better word, when there is incongruence between the systems, and/or when IK will be “outside” the community or used by “external” interests (e.g., as knowledge(s) are being translated to decisions (Kobluk et al. 2024)). Indigenous Peoples are more likely, we think, to have training in both—they have lived experience and obtain some Western education. And, in the education system, Western science has been the only science taught. Non‐Indigenous Peoples more often do not have lived experience of Indigenous ways of knowing, and until now have rarely learned about IK. Weaving becomes important at different scales and levels when boards or committees are making decisions rather than individuals; this is when issues tend to arise.

### 4.2 Some Other Considerations

Although we extrapolate our findings to principles for braiding, we want to caution against pan‐Indigenizing our findings. In this work, we interviewed 40 people with membership in 12 communities belonging to six Nations/culture groups, while the case studies generally span Canada. The terrestrial case studies are largely located in modern treaty areas, but the freshwater studies and coastal‐marine studies extend the distribution of studies extensively. Many voices are not represented, though we hope that it is a strength of the work that we combined oral and written stories.

Additionally, the vast majority of the written documents reviewed have been written from a Western scientific perspective. Within this paradigm, it is only recently that IK are being considered more equally with Western science, and that co‐development of research and equal participation of the people involved has been at the fore (Alexander et al. 2021, 2019; Provencher et al., n.d.). Tangential to this, coding is a Western reductionist approach. In some ways, we may have forced categories where approaches fall more in the gray zone. In particular, coding methods as Western versus Indigenous can be very challenging because they can be intensely intertwined in the case studies. Lastly, our literature base is likely biased to works with terminology and content that has historically been palatable for Western‐biased reviewers (e.g., writing about dreamscapes and science may not have been published historically).

Also surrounding coding, we developed the codes for roles for IK and WS based on screening ~50 papers that brought together IK and WS for biodiversity work. Initially, we did not code roles for IK and WS for data collection because we thought the methods for IK collation and WS data collection would be an equivalent type of information. However, it became clear that they are complementary information. We retroactively coded roles at data collection, but further work could be done to better characterize roles at data collection. For example, in some cases, only one code was included for roles, but that both IK and WS were used at a given stage was also coded.

While intergenerational knowledge transfer did not correlate with either the extent of braiding or engagement, this may be because it was reported so rarely in the peer‐reviewed case studies. If it was discussed more, a trend may have been evident. As shown in Figure S2, whether gender representation was discussed (demographics_gender) is correlated with the year published. In fact, it is the only variable correlated with the year published. It may be the case that intergenerational knowledge transfer will be discussed more in coming years with increased Indigenous‐led work and better understanding amongst other researchers of its importance, as seen in the following national and international works (Bulmer et al. 2024; Esquible et al. 2024; Reo et al. 2017).

Lastly, we have taken you through a journey, starting with what Indigenous and Western knowledges mean to Indigenous Peoples, and what Indigenous Peoples' feel their roles should be in environmental research and monitoring. We included quotes and stories from the people engaged in doing this work in part because we feel that the foundations shared in those words can help you to better understand the ultimate outcomes of our braided work. After all, how can a reader understand why monitoring needs to be led by IK without understanding what IK is to the people who have spoken about it?

### 4.3 Looking to the Future, Implications for Policy and Practice

Ongoing funding is needed to support capacity‐sharing within Indigenous communities, organizations, and project teams, in order to build and maintain the relationships and capacity needed for respectful knowledge braiding projects. Multi‐year funding to support language revitalization and lands‐based activities is key to capacity development within communities for the implementation of biodiversity monitoring within local contexts and knowledge systems. This funding includes a need to shift power and resources into Indigenous communities so that more work can be directed by and for them. It is necessary that timelines for biodiversity programs are flexible to allow for (1) relationship‐building, (2) meaningful team engagement throughout the research project, and (3) space for the dynamics of multiple knowledge systems to be considered in the context of each other, which in and of itself can differ from project to project. While there has been some work to develop IK frameworks within the Government of Canada (Assessment Agency of Canada, Transport Canada, Canada Energy Regulator, and Fisheries and Oceans Canada 2019; DFO 2019b), to our knowledge, no department currently has a framework or clear messaging that is publicly accessible (Hill et al. 2019); given the adoption of the United Nations Declaration Act (UNDA), frameworks are needed. Our indicators reflect that researchers or project leaders hoping to do this work well could focus on engaging with communities early and often, ensuring that work is relevant to community needs, paying special attention to data sovereignty and governance, and paying attention to the accessibility of the information to communities if they wish to braid at most stages of their work. In so doing, the FAIR, CARE, and OCAP principles must be upheld. IK needs to be findable, accessible, interoperable, and reusable (FAIR) within the context of CARE. That is, there must also be collective benefit to those who shared the information, Indigenous People must have the authority to control their data, and responsibility to protect their data and knowledge for future generations, and ethics must be prioritized (CARE) (Russo Carroll et al. 2021). Additionally, project leads must uphold ownership, control, access, and possession of the information shared (OCAP (https://fnigc.ca/ocap‐training/)).

Through our analysis, it is not possible to know whether it was communities or researchers that drove the level of engagement seen in the case studies. However, interview participants consistently voiced the need for more comprehensive inclusion. Within Canada, the implementation of the UNDA may create more engagement—however, there are some significant hurdles in Canada (and other countries) to meaningfully implementing UNDRIP (Côté et al. 2024). Issues surrounding Indigenous self‐determination and free prior and informed consent (FPIC) are very common. Both issues intersect significantly with braiding knowledges in biodiversity work, where, at their most basic, FPIC enforces the need for engagement before and throughout project development, including in decision‐making, and self‐determination acknowledges and supports Indigenous leadership in research governance (Jessen et al. 2022). David‐Chavez and Gavin (2018) and Ibbett and Brittain (2019) provide clear guidance on how to uphold these tenets, and we included much of their guidance in our coding and analysis. Upholding the indicators that we found should help researchers follow these principles.

In addition, we have addressed primarily research and monitoring in this work because this forms the foundation for evidence‐based management and decision‐making. However, decision‐making resulting from research often happens in different spaces, with different people, and at different times. So much important research is not given enough weight at the decision‐making table or does not make it to the table if connections are not made between researchers or research products and decision‐makers. A further work examining patterns and processes of braiding in management and decision‐making has been completed to help understand some of these challenges and build the paradigm shifts that are needed in colonial governments and institutions (Nishima‐Miller et al. 2025).

As discussed in this paper, there can be hesitation for both Indigenous and non‐Indigenous Peoples to reach out to each other to work collaboratively on environmental projects, likely due to historical and contemporary injustices and inequalities. However, shining through this work are both a desire and a need to connect, but to do so in a respectful way. People who are not Indigenous must take the time to learn and participate fully in our shared history and current relationships.

Oh, definitely [it is appropriate for non‐Indigenous People to hear and learn Indigenous stories that tell of Indigenous laws], because one of the responsibilities when it comes to knowledge acquisition is sharing that knowledge…

I feel that when non‐Indigenous People actually participate in our ceremonies, they start to have a better understanding of who we actually are. —Sue Chiblow, participant and co‐author

## Author Contributions

**Ella Bowles:** conceptualization (lead), data curation (lead), formal analysis (lead), funding acquisition (equal), investigation (lead), methodology (equal), project administration (lead), resources (equal), supervision (lead), validation (lead), visualization (equal), writing – original draft (lead), writing – review and editing (lead). **Dominique A. Henri:** conceptualization (equal), data curation (equal), formal analysis (supporting), funding acquisition (equal), investigation (supporting), methodology (equal), supervision (supporting), validation (supporting), writing – review and editing (equal). **Jennifer F. Provencher:** conceptualization (equal), data curation (equal), formal analysis (supporting), funding acquisition (equal), investigation (equal), methodology (equal), supervision (supporting), validation (equal), writing – review and editing (equal). **Steven M. Alexander:** conceptualization (equal), data curation (supporting), formal analysis (supporting), investigation (supporting), methodology (equal), validation (supporting), writing – review and editing (equal). **Nicola E. Love:** data curation (equal), formal analysis (lead), methodology (equal), software (equal), validation (equal), visualization (equal), writing – original draft (equal), writing – review and editing (equal). **Jade Steel:** conceptualization (supporting), data curation (equal), investigation (equal), methodology (supporting), validation (supporting), writing – review and editing (supporting). **Carmen Chelick:** conceptualization (supporting), data curation (equal), investigation (equal), methodology (supporting), validation (supporting), writing – review and editing (supporting). **Junaid S. Khan:** data curation (supporting), formal analysis (equal), validation (supporting), writing – review and editing (supporting). **Jessica J. Taylor:** conceptualization (equal), data curation (equal), methodology (equal), software (equal), validation (equal), writing – review and editing (supporting). **Britney Zacharuk:** data curation (equal), formal analysis (equal), visualization (supporting), writing – review and editing (supporting). **Alana Wilcox:** data curation (equal), investigation (equal), validation (supporting), writing – review and editing (supporting). **Oscar Hartman Davies:** data curation (equal), investigation (equal), validation (supporting), writing – review and editing (supporting). **Deborah McGregor:** conceptualization (supporting), funding acquisition (supporting), investigation (supporting), methodology (equal), validation (supporting), writing – review and editing (supporting). **Susan Chiblow:** conceptualization (supporting), funding acquisition (supporting), investigation (supporting), validation (supporting), writing – review and editing (supporting). **Steven J. Cooke:** funding acquisition (supporting), resources (equal), supervision (equal), writing – review and editing (supporting). **Adam T. Ford:** conceptualization (equal), funding acquisition (equal), supervision (equal), validation (supporting), writing – review and editing (supporting). **Jesse N. Popp:** conceptualization (equal), funding acquisition (equal), methodology (supporting), supervision (equal), validation (supporting), writing – review and editing (supporting).

## Conflicts of Interest

The authors declare no conflicts of interest.

## Supporting information


**Data S1:** ece372358‐sup‐0001‐supinfo.docx.

**Data S2:** ece372358‐sup‐0002‐supinfo.csv.

**Data S3:** ece372358‐sup‐0003‐Bowles et al_Code_practices for braid‐SumStats_Supplementary_S3.R.

## Data Availability

All coded information from the literature as well as analysis scripts are available as Supporting Information (Files S2 and S3, respectively). Full interview/sharing circle transcripts will not be made publicly available because it is not appropriate for other investigators to utilize the information without informed consent of the participants.

## References

[ece372358-bib-0001] Alexander, S. M. , J. F. Provencher , D. A. Henri , et al. 2021. “Bridging Indigenous and Western Sciences in Freshwater Research, Monitoring, and Management in Canada.” Ecological Solutions and Evidence 2, no. 3: e12085.

[ece372358-bib-0002] Alexander, S. M. , J. F. Provencher , D. A. Henri , J. J. Taylor , and S. J. Cooke . 2019. “Bridging Indigenous and Science‐Based Knowledge in Coastal‐Marine Research, Monitoring, and Management in Canada: A Systematic Map Protocol.” Environmental Evidence 8, no. 1: 15. 10.1186/s13750-019-0159-1.

[ece372358-bib-0003] Arsenault, R. , S. Diver , D. McGregor , A. Witham , and C. Bourassa . 2018. “Shifting the Framework of Canadian Water Governance Through Indigenous Research Methods: Acknowledging the Past With an Eye on the Future.” Water (Basel) 10, no. 1: 49.

[ece372358-bib-0004] Artelle, K. , M. Adams , H. Bryan , et al. 2021. “Decolonial Model of Environmental Management and Conservation: Insights From Indigenous‐Led Grizzly Bear Stewardship in the Great Bear Rainforest.” Ethics, Policy & Environment 24, no. 3: 283–323.

[ece372358-bib-0005] Assessment Agency of Canada, Transport Canada, Canada Energy Regulator, and Fisheries and Oceans Canada . 2019. Indigenous Knowledge Policy Framework for Proposed Project Reviews and Regulatory Decisions (p. 9). Governement of Canada. https://www.canada.ca/en/services/environment/conservation/assessments/environmental‐reviews/environmental‐assessment‐processes/discussion‐paper‐development‐indigenous‐knowledge‐policy‐framework.html.

[ece372358-bib-0006] Bartlett, C. , M. Marshall , and A. Marshall . 2012. “Two‐Eyed Seeing and Other Lessons Learned Within a Co‐Learning Journey of Bringing Together Indigenous and Mainstream Knowledges and Ways of Knowing.” Journal of Environmental Studies and Sciences 2, no. 4: 331–340.

[ece372358-bib-0007] Battiste, M. 2002. Indigenous Knowledge and Pedagogy in First Nations Education: A Literature Review With Recommendations. *A report prepared for the* National Working Group on Education. Indian and Northern Affairs Canada.

[ece372358-bib-0008] BC Government . 2014. “Environmental Stewardship Initiative.” https://www2.gov.bc.ca/gov/content/environment/natural‐resource‐stewardship/consulting‐with‐first‐nations/collaborative‐stewardship‐bc/environmental‐stewardship‐initiative.

[ece372358-bib-0009] Berkes, F. 2012. Sacred Ecology. Routledge.

[ece372358-bib-0010] Bowles, E. , H.‐B. Jeon , K. Marin , P. MacLeod , and D. J. Fraser . 2022. “Freshwater Fisheries Monitoring in Northern Ecosystems Using Indigenous Ecological Knowledge, Genomics, and Life History: Insights for Community Decision‐Making.” Facets 7, no. 1: 1214–1243.

[ece372358-bib-0011] Bowles, E. , J. S. Khan , A. K. Menzies , A. T. Ford , D. McGregor , and J. N. Popp . 2023. Perspectives on if, When and How to Braid Indigenous Knowledges and Western Science in Biodiversity and Environmental Research, Monitoring and Management. A report from the Weaving Indigenous Knowledge Systems and Western Science Towards Conservation and Management of Wildlife and the Environment project . Virtual.

[ece372358-bib-0012] Bowles, E. , A. K. Menzies , J. S. Khan , A. T. Ford , D. McGregor , and J. N. Popp . 2023. Cultural Keystone Species, Environmental Concerns and Future Research Priorities. A report from the Weaving Indigenous Knowledge Systems and Western Science Towards Conservation and Management of Wildlife and the Environment project . Virtual.

[ece372358-bib-0013] Brundtland, G. H. 1987. Report of the World Commission on Environment and Development: ‘Our Common Future’. UN.

[ece372358-bib-0014] Bulmer, R. , K. Paul‐Burke , M. Ranapia , et al. 2024. “Weaving Indigenous and Western Ecological Knowledge to Enhance Environmental Sustainability.” Ocean and Coastal Management 258, no. 1: 107402.

[ece372358-bib-0015] Buxton, R. T. , J. R. Bennett , A. J. Reid , et al. 2021. “Key Information Needs to Move From Knowledge to Action for Biodiversity Conservation in Canada.” Biological Conservation 256: 108983.

[ece372358-bib-0016] Cajete, G. 2000. Native Science: Natural Laws of Interdependence. Clear Light Publishers.

[ece372358-bib-0019] Canadian Environmental Protection Act (CEPA), Pub. L. No. S.C. c.33 . 1999. https://laws‐lois.justice.gc.ca/eng/acts/c‐15.31/FullText.html.

[ece372358-bib-0020] CCIRA . n.d. “Central Coast Indigenous Resource Alliance.” Accessed 1 January 2021. https://www.ccira.ca/k.

[ece372358-bib-0021] Chiblow, S. , and World Health Organization . 2024. The Languages, Cultures, Wisdom, and Scientific and Technical Knowledge of Indigenous Peoples Within the Context of the Climate Crisis. In State of the World's Indigenous Peoples (Vols. 1–6). United Nations.

[ece372358-bib-0022] Colleges and Institutes Canada . 2024. By the Numbers: Indigenous Post‐Secondary Education in Canada. Colleges and Institutes Canada. https://www.collegesinstitutes.ca/by‐the‐numbers‐indigenous‐post‐secondary‐education‐in‐canada/.

[ece372358-bib-0023] Côté, I. , A. Grant , A. Islam , V. McLean , M. I. Mitchell , and D. Panagos . 2024. “The Global Implementation of UNDRIP: A Thematic Review.” International Journal of Human Rights 29, no. 2: 306–330.

[ece372358-bib-0024] Council of the Haida Nation . 2018. “Gwaii Haanas Gina'Waadluxan Kilguhlaga Land‐Sea‐People Management Plan.”

[ece372358-bib-0025] Craft, A. 2018. “Navigating Our Ongoing Sacred Legal Relationship With Nibi (Water).” UNDRIP Implementation: More Reflections on the Braiding of International, Domestic and Indigenous Laws 53 62.

[ece372358-bib-0026] Daigle, M. 2016. “Awawanenitakik: The Spatial Politics of Recognition and Relational Geographies of Indigenous Self‐Determination.” Canadian Geographies/Géographies Canadiennes 60, no. 2: 259–269.

[ece372358-bib-0027] David‐Chavez, D. M. , and M. C. Gavin . 2018. “A Global Assessment of Indigenous Community Engagement in Climate Research.” Environmental Research Letters 13: 123005.

[ece372358-bib-0028] Dawson, N. , B. Coolsaet , E. Sterling , et al. 2021. “The Role of Indigenous Peoples and Local Communities in Effective and Equitable Conservation.” Ecology and Society 26, no. 3: 19.

[ece372358-bib-0029] DFO . 2019a. “Fisheries Policies and Frameworks.” http://www.dfo‐mpo.gc.ca/reports‐rapports/regs/policies‐politiques‐eng.htm.

[ece372358-bib-0030] DFO . 2019b. Reconciliation Strategy. Government of Canada, Department of Fisheries and Oceans. http://www.dfo‐mpo.gc.ca/fisheries‐peches/aboriginal‐autochtones/reconciliation‐eng.html.

[ece372358-bib-0031] Dickson‐Hoyle, S. , R. E. Ignace , M. B. Ignace , S. M. Hagerman , L. D. Daniels , and K. Copes‐Gerbitz . 2022. “Walking on Two Legs: A Pathway of Indigenous Restoration and Reconciliation in Fire‐Adapted Landscapes.” Restoration Ecology 30, no. 4: e13566.

[ece372358-bib-0032] Eckert, L. E. , N. X. Claxton , C. Owens , et al. 2020. “Indigenous Knowledge and Federal Environmental Assessments in Canada: Applying Past Lessons to the 2019 Impact Assessment Act.” Facets 5, no. 1: 67–90.

[ece372358-bib-0033] Eisner, W. R. , J. Jelacic , C. J. Cuomo , C. Kim , K. M. Hinkel , and D. Del Alba . 2012. “Producing an Indigenous Knowledge Web GIS for Arctic Alaska Communities: Challenges, Successes, and Lessons Learned.” Transactions in GIS 16, no. 1: 17–37.

[ece372358-bib-0035] Esquible, J. , A. Hoffman , D. Lowrey , et al. 2024. “Aulukluki Neqkat: Centering Care of Salmon and Relational Research in Indigenous Fisheries in the Kuskokwim River, Alaska.” Arctic Science 10, no. 2: 349–371.

[ece372358-bib-0036] Fisher, R. A. 1958. Statistical Methods for Research Workers. Oliver and Boyd.

[ece372358-bib-0037] Gagnon, C. , and D. Berteaux . 2009. “Integrating Traditional Ecological Knowledge and Ecological Science: A Question of Scale.” Ecology and Society 14, no. 2: art19.

[ece372358-bib-0038] Gallant, M. , E. Bowles , H. Patterson , and J. N. Popp . 2020. Anishinaabe Knowledge, Concerns, and Priorities Related to Climate Change: A Report From the ‘Connecting Guardians in a Changing World’ Workshop. Magnetawan First Nation, Mount Allison University.

[ece372358-bib-0039] Garnett, S. T. , N. D. Burgess , J. E. Fa , et al. 2018. “A Spatial Overview of the Global Importance of Indigenous Lands for Conservation.” Nature Sustainability 1, no. 7: 369–374.

[ece372358-bib-0040] Grand Council of the Crees (GCC) . 2011. “Cree Vision of Plan Nord.” https://www.cngov.ca/wp‐content/uploads/2018/03/cree‐vision‐of‐plan‐nord.pdf.

[ece372358-bib-0041] Haddaway, N. R. , M. Land , and B. Macura . 2017. “‘A Little Learning is a Dangerous Thing’: A Call for Better Understanding of the Term ‘Systematic Review’.” Environment International 99: 356–360. 28041639 10.1016/j.envint.2016.12.020

[ece372358-bib-0042] Haddaway, N. R. , B. Macura , P. Whaley , and A. S. Pullin . 2018. “ROSES RepOrting Standards for Systematic Evidence Syntheses: Pro Forma, Flow‐Diagram and Descriptive Summary of the Plan and Conduct of Environmental Systematic Reviews and Systematic Maps.” Environmental Evidence 7, no. 1: 1–8.

[ece372358-bib-0043] Haddaway, N. R. , and M. J. Westgate . 2019. “Predicting the Time Needed for Environmental Systematic Reviews and Systematic Maps.” Conservation Biology 33, no. 2: 434–443. 30285277 10.1111/cobi.13231

[ece372358-bib-0044] Henri, D. A. , J. F. Provencher , E. Bowles , et al. 2021. “Weaving Indigenous Knowledge Systems and Western Sciences in Terrestrial Research, Monitoring and Management in Canada: A Protocol for a Systematic Map.” Ecological Solutions and Evidence 2, no. 2: e12057.

[ece372358-bib-0045] Hessami, M. A. , E. Bowles , J. N. Popp , and A. T. Ford . 2021. “Indigenizing the North American Model of Wildlife Conservation.” Facets 6, no. 1: 1285–1306.

[ece372358-bib-0046] Hill, C. J. , R. Schuster , and J. R. Bennett . 2019. “Indigenous Involvement in the Canadian Species at Risk Recovery Process.” Environmental Science & Policy 94: 220–226.

[ece372358-bib-0047] Housty, W. G. , A. Noson , G. W. Scoville , et al. 2014. “Grizzly Bear Monitoring by the Heiltsuk People as a Crucible for First Nation Conservation Practice.” Ecology and Society 19, no. 2: art70.

[ece372358-bib-0048] Huntington, H. , T. Callaghan , S. Fox , and I. Krupnik . 2004. “Matching Traditional and Scientific Observations to Detect Environmental Change: A Discussion on Arctic Terrestrial Ecosystems.” Ambio 33, no. 7: 18–23. 15575178

[ece372358-bib-0049] Ibbett, H. , and S. Brittain . 2019. “Conservation Publications and Their Provisions to Protect Research Participants.” Conservation Biology 34, no. 1: 80–92. 31016794 10.1111/cobi.13337 PMC7028057

[ece372358-bib-0050] IPBES . 2019. Summary for Policymakers of the Global Assessment Report on Biodiversity and Ecosystem Services of the Intergovernmental Science‐Policy Platform on Biodiversity and Ecosystem Services, edited by S. Díaz , J. Settele , E. S. Brondízio , et al., 56. IPBES Secretariat.

[ece372358-bib-0051] Jacobs, D. , and V. Lytwyn . 2020. “Naagan Ge Bezhig Emkwaan: A Dish With One Spoon Reconsidered.” Ontario History 112, no. 2: 191–210.

[ece372358-bib-0052] Jessen, T. D. , N. C. Ban , N. X. Claxton , and C. T. Darimont . 2022. “Contributions of Indigenous Knowledge to Ecological and Evolutionary Understanding.” Frontiers in Ecology and the Environment 20, no. 2: 93–101.

[ece372358-bib-0053] Jones, B. L. , R. O. Santos , W. R. James , et al. 2024. “New Directions for Indigenous and Local Knowledge Research and Application in Fisheries Science: Lessons From a Systematic Review.” Fish and Fisheries 25: 647–671.

[ece372358-bib-0054] Joseph, R. P. 2018. 21 Things You May Not Know About the Indian Act: Helping Canadians Make Reconciliation With Indigenous Peoples a Reality (1st ed.). Indigenous relations press.

[ece372358-bib-0055] Kimmerer, R. W. 2013. Braiding Sweetgrass: Indigenous Wisdom, Scientific Knowledge and the Teachings of Plants. Milkweed Editions.

[ece372358-bib-0056] Kobluk, H. M. , A. K. Salomon , A. T. Ford , et al. 2024. “Relational Place‐Based Solutions for Environmental Policy Misalignments.” Trends in Ecology & Evolution 39: 217–220. 38278702 10.1016/j.tree.2024.01.001

[ece372358-bib-0057] Kruskal, W. H. , and W. A. Wallis . 1952. “Use of Ranks in One‐Criterion Variance Analysis.” Journal of the American Statistical Association 47, no. 260: 583–621.

[ece372358-bib-0058] Levac, L. , L. McMurtry , D. Stienstra , G. Baikie , C. Hanson , and D. Mucina . 2018. Learning Across Indigenous and Western Knowledge Systems and Intersectionality: Reconciling Social Science Research Approaches (Unpublished SSHRC Knowledge Synthesis Report), p. 40. University of Guelph, Government of Canada.

[ece372358-bib-0059] Lumivero . 2018. “NVivo (Version 12)” [Computer Software]. https://www.qsrinternational.com/nvivo‐qualitative‐data‐analysis‐software/home.

[ece372358-bib-0060] Mallory, M. L. , A. J. Fontaine , J. A. Akearok , and V. H. Johnston . 2006. “Synergy of Local Ecological Knowledge, Community Involvement and Scientific Study to Develop Marine Wildlife Areas in Eastern Arctic Canada.” Polar Record 42, no. 3: 205–216.

[ece372358-bib-0061] McGregor, D. 2004. “Traditional Ecological Knowledge and Sustainable Development Towards Coexistence.” IDRC. Available in: En: Er‐64525‐201‐Do_Topic. Html.

[ece372358-bib-0062] McGregor, D. 2009. “Honouring Our Relations: An Anishnaabe Perspective.” Speaking for Ourselves: Environmental Justice in Canada 27: 27–41.

[ece372358-bib-0063] McGregor, D. 2014. “Traditional Knowledge and Water Governance: The Ethic of Responsibility.” AlterNative: An International Journal of Indigenous Peoples 10, no. 5: 493–507.

[ece372358-bib-0064] McGregor, D. 2018. “From ‘Decolonized’ to Reconciliation Research in Canada: Drawing From Indigenous Research Paradigms.” ACME: An International Journal for Critical Geographies 17, no. 3: 810–831.

[ece372358-bib-0065] McGregor, D. 2021. “Indigenous Knowledge Systems in Environmental Governance in Canada.” KULA: Knowledge Creation, Dissemination, and Preservation Studies 5, no. 1: 1–10.

[ece372358-bib-0066] McGregor, D. 2023. “Indigenous Sustainable Development: Shaping Our Future.” In The Palgrave Handbook of Global Sustainability, 1041–1053. Springer.

[ece372358-bib-0067] McGregor, D. , S. Whitaker , and M. Sritharan . 2020. “Indigenous Environmental Justice and Sustainability.” Current Opinion in Environmental Sustainability 43: 35–40.

[ece372358-bib-0068] Menzies, A. K. , E. Bowles , M. Gallant , et al. 2022. ““I See My Culture Starting to Disappear”: Anishinaabe Perspectives on the Sociecological Impacts of Climate Change and Future Research Needs.” Facets 7, no. 1: 509–527. 10.1139/facets-2021-006.

[ece372358-bib-0069] Menzies, A. K. , E. Bowles , J. S. Khan , D. McGregor , A. T. Ford , and J. N. Popp . 2022. Caring for the Land: Indigenous Practices, Community Values, Changes Over Time, and Building Values‐Based Environmental Initiatives. A report from the Weaving Indigenous Knowledge Systems and Western Science Towards Conservation and Management of Wildlife and the Environment project . Virtual.

[ece372358-bib-0070] Menzies, A. K. , E. Bowles , D. McGregor , A. T. Ford , and J. N. Popp . 2024. “Sharing Indigenous Values, Practices and Priorities as Guidance for Transforming Human–Environment Relationships.” People and Nature 6, no. 5: 2109–2125.

[ece372358-bib-0071] Mercier, O. R. , and A.‐M. Jackson . 2023. “Indigenous Discourse in the Mainstream: The Case of “Matuauranga and Science” in New Zealand Science Review.” In Race and Sociocultural Inclusion in Science Communication: Innovation, Deconolisation, and Transformation, 130–146. Policy Press.

[ece372358-bib-0072] Migratory Birds Convention Act (MBCA), S.C . 1994. c. 22. https://laws‐lois.justice.gc.ca/eng/acts/M‐7.01/index.html.

[ece372358-bib-0073] Mitchell, H. 2011. Working With Elders and Indigenous Knowledge Systems: A Reader and Guide for Places of Higher Learning. J Carlton Publishing.

[ece372358-bib-0076] *M’sɨt* *No'kmaq* , A. Marshall , K. F. Beazley , et al. 2021. “‘Awakening the Sleeping Giant’: Re‐Indigenization Principles for Transforming Biodiversity Conservation in Canada and Beyond.” Facets 6, no. 1: 839–869.

[ece372358-bib-0075] Nishima‐Miller, J. , L. R. Johnson , J. F. Provencher , et al. 2025. “Bridging Indigenous Knowledge Systems and Western Science for the Co‐Management of Wildlife in Canada: A Systematic Review.” Environmental Reviews 33: 1–19.

[ece372358-bib-0078] Okanagan Nation Alliance . 2021. ks͜ kəɬqayxwntim iʔ siwɬkw: We Will Protect the Water. https://syilx.org/wp‐content/uploads/2022/07/FINAL‐Water‐Strategy_2022‐Edition.pdf.

[ece372358-bib-0079] Parks Canada . 2019. “Mapping Change: Fostering a Culture of Reconciliation Within Parks Canada.” https://www.pc.gc.ca/en/agence‐agency/aa‐ia/reconciliation.

[ece372358-bib-0080] Parlee, B. , M. Manseau , and Łutsël K'é Dene First Nation . 2005. “Using Traditional Knowledge to Adapt to Ecological Change: Denésǫłıné Monitoring of Caribou Movements.” Arctic 59, no. 1: 26–37.

[ece372358-bib-0081] Patterson, H. , E. Bowles , and J. N. Popp . 2020. Environmental and Sociocultural Impacts of Glyphosate‐Based Herbicides Used in Forestry: Perspectives From Indigenous Knowledge and Western Science (p. 63). Magnetawan First Nation.

[ece372358-bib-0082] Pedersen, C. , M. Otokiak , I. Koonoo , et al. 2020. “ScIQ: An Invitation and Recommendations to Combine Science and Inuit Qaujimajatuqangit for Meaningful Engagement of Inuit Communities in Research.” Arctic Science 6, no. 3: 326–339.

[ece372358-bib-0083] Plummer, R. , D. de Grosbois , D. Armitage , and R. C. de Loë . 2013. “An Integrative Assessment of Water Vulnerability in First Nation Communities in Southern Ontario, Canada.” Global Environmental Change 23, no. 4: 749–763.

[ece372358-bib-0084] Polfus, J. L. , K. Heinemeyer , and M. Hebblewhite . 2014. “Comparing Traditional Ecological Knowledge and Western Science Woodland Caribou Habitat Models.” Journal of Wildlife Management 78, no. 1: 112–121.

[ece372358-bib-0085] Polfus, J. L. , M. Manseau , D. Simmons , et al. 2016. “Łeghágots' enetę (Learning Together): The Importance of Indigenous Perspectives in the Identification of Biological Variation.” Ecology and Society 21, no. 2: 18.

[ece372358-bib-0086] Prosper, K. , L. J. McMillan , A. A. Davis , and M. Moffitt . 2011. “Returning to Netukulimk: Mi'kmaq Cultural and Spiritual Connections With Resource Stewardship and Self‐Governance.” International Indigenous Policy Journal 2, no. 4: 7.

[ece372358-bib-0087] Provencher, J. F. , E. Bowles , D. A. Henri , et al. n.d. “Weaving Indigenous Knowledge Systems and Western Sciences in Terrestrial Research, Monitoring and Management in Canada: A Systematic Map.” Submitted.

[ece372358-bib-0088] Pullin, A. S. , G. Frampton , B. Livoreil , and G. Petrokofsky . 2018. “Collaboration for Environmental Evidence (Vol. 5).” www.environmentalevidence.org/information‐for‐authors.

[ece372358-bib-0089] R Core Team . 2022. “R: A Language and Environment for Statistical Computing (Version 4.2.0).” [Computer Software].

[ece372358-bib-0090] Rathwell, K. J. , A. K. Menzies , L. R. Johnson , et al. 2025. “A Growing Tree Metaphor: Identifying and Reflecting on 26 Action Items for Ethical Bridging of Indigenous and Western Knowledge Systems in Biodiversity Research and Monitoring.” Ecology and Society 30, no. 2: 30. 10.5751/ES-15946-300230.

[ece372358-bib-0091] Reid, A. J. , L. E. Eckert , J. Lane , et al. 2021. ““Two‐Eyed Seeing”: An Indigenous Framework to Transform Fisheries Research and Management.” Fish and Fisheries 22, no. 2: 243–261.

[ece372358-bib-0092] Reid, A. J. , D. A. McGregor , A. K. Menzies , L. E. Eckert , C. M. Febria , and J. N. Popp . 2024. “Ecological Research ‘in a Good Way’ Means Ethical and Equitable Relationships With Indigenous Peoples and Lands.” Nature Ecology & Evolution 8, no. 4: 595–598. 38225427 10.1038/s41559-023-02309-0

[ece372358-bib-0093] Reo, N. J. , K. P. Whyte , D. McGregor , M. Smith , and J. F. Jenkins . 2017. “Factors That Support Indigenous Involvement in Multi‐Actor Environmental Stewardship.” AlterNative: An International Journal of Indigenous Peoples 13, no. 2: 58–68.

[ece372358-bib-0094] Riedlinger, D. , and F. Berkes . 2001. “Contributions of Traditional Knowledge to Understanding Climate Change in the Canadian Arctic.” Polar Record 37, no. 203: 315–328.

[ece372358-bib-0095] Russo Carroll, S. , E. Herczog , M. Hudson , K. Russell , and S. Stall . 2021. Operationalizing the CARE and F.AIR Principles for Indigenous Data Futures. Scientific Data. 10.1038/s41597-021-00892-0 PMC8052430 33863927

[ece372358-bib-0096] Saldana, J. 2021. The Coding Manual for Qualitative Researchers. SAGE Publications Ltd.

[ece372358-bib-0097] SARA . 2002. “Species at Risk Act, Pub. L. No. S.C. 2002, c. 29.”

[ece372358-bib-0099] Starblanket, G. , and H. Stark . 2018. Towards a Relational Paradigm—Four Points for Consideration: Knowledge, Gender, Land, and Modernity. University of Toronto Press.

[ece372358-bib-0100] Stefanelli, R. D. , H. Castleden , S. L. Harper , D. Martin , A. Cunsolo , and C. Hart . 2017. “Experiences With Integrative Indigenous and Western Knowledge in Water Research and Management: A Systematic Realist Review of Literature From Canada, Australia, New Zealand, and the United States.” Environmental Reviews 25, no. 3: 323–333.

[ece372358-bib-0101] Stern, E. R. , and M. M. Humphries . 2022. “Interweaving Local, Expert, and Indigenous Knowledge Into Quantitative Wildlife Anlyses: A Systematic Review.” Biological Conservation 266: 109444.

[ece372358-bib-0102] Tengö, M. , E. S. Brondizio , T. Elmqvist , P. Malmer , and M. Spierenburg . 2014. “Connecting Diverse Knowledge Systems for Enhanced Ecosystem Governance: The Multiple Evidence Base Approach.” Ambio 43, no. 5: 579–591. 24659474 10.1007/s13280-014-0501-3 PMC4132468

[ece372358-bib-0103] Tengö, M. , R. Hill , P. Malmer , et al. 2017. “Weaving Knowledge Systems in IPBES, CBD and Beyond—Lessons Learned for Sustainability.” Current Opinion in Environmental Sustainability 26, no. 27: 17–25.

[ece372358-bib-0104] Thompson, K.‐L. , T. Lantz , and N. Ban . 2020. “A Review of Indigenous Knowledge and Participation in Environmental Monitoring.” Ecology and Society 25, no. 2: art10.

[ece372358-bib-0105] Thornton, T. F. , and A. M. Scheer . 2012. “Collaborative Engagement of Local and Traditional Knowledge and Science in Marine Environments: A Review.” Ecology and Society 17, no. 3: art8.

[ece372358-bib-0106] Truth and Reconciliation Commission of Canada . 2015. Honouring the Truth, Reconciling for the Future: Summary of the Final Report of the Truth and Reconciliation Commission of Canada (Vol. 1). Library and Archives Canada. https://nctr.ca/assets/reports/Final%20Reports/Executive_Summary_English_Web.pdf.

[ece372358-bib-0107] UN general assembly . 2007. United Nations Declaration on the Rights of Indigenous Peoples: Resolution/Adopted by the General Assembly (No. A/RES/61/295). UN General Assembly. https://www.un.org/development/desa/indigenouspeoples/wp‐content/uploads/sites/19/2018/11/UNDRIP_E_web.pdf.

[ece372358-bib-0108] United Nations Declaration on the Rights of Indigenous Peoples Act (UNDA), Pub. L. No. S,C, c. 14 . 2021. https://www.laws‐lois.justice.gc.ca/eng/acts/u‐2.2/page‐1.html.

[ece372358-bib-0109] Webb, T. M. 2024. What's the Matter With CANZUS? Understanding Canada, Australia, New Zealand, and the United States' UNDRIP Reversal [MSc]. University of Chicago.

[ece372358-bib-0110] Wilson, N. J. , and J. Inkster . 2018. “Respecting Water: Indigenous Water Governance, Ontologies, and the Politics of Kinship on the Ground.” Environment and Planning E: Nature and Space 1, no. 4: 516–538.

[ece372358-bib-0111] Wong, C. , K. Ballegooyen , L. Ignace , M. J. Johnson , and H. Swanson . 2020. “Towards Reconciliation: 10 Calls to Action to Natural Scientists Working in Canada.” Facets 5, no. 1: 769–783.

[ece372358-bib-0112] Youdelis, M. 2016. ““They Could Take You out for Coffee and Call It Consultation!”: The Colonial Antipolitics of Indigenous Consultation in Jasper National Park.” Environment and Planning A: Economy and Space 48, no. 7: 1374–1392.

[ece372358-bib-0113] Yua, E. , J. Raymond‐Yakoubian , R. Aluaq Daniel , and C. Behe . 2022. “A Framework for Co‐Production of Knowledge in the Context of Arctic Research.” Ecology and Society 27, no. 1: 34. 10.5751/ES-12960-270134.

