# Supplementary material for: Practices for Braiding Indigenous Knowledges and Western Sciences for Research and Monitoring of Biodiversity in Canada
Source: Ecol Evol. 2026 Jan 22;16(1):e72358. doi: 10.1002/ece3.72358 (PMC12828175; doi:10.1002/ece3.72358)
Supplement: Supplementary file 1 — Data S1: ece372358‐sup‐0001‐supinfo.docx. [file ECE3-16-e72358-s001.docx]

**Practices for braiding Indigenous knowledges and Western sciences for research and monitoring of biodiversity in Canada**

Bowles et al.

**Supplementary material**

Indigenous knowledges

**Supplementary Table S1:** Interview and sharing circle questions

1. *What do you think people from outside your community can do better to work with your community to help take care of plants, animals and the environment?
2. Have you heard of the term Traditional Knowledge or Traditional Ecological Knowledge or Indigenous Knowledge Systems?
   1. Are there other words that you use for it? ---note for interviewer: if you are speaking to a language speaker ask ‘How do you explain it in your language?’
   2. What does ‘this knowledge’ mean to you?
3. What is the role of ‘this knowledge’ in taking care of (research, monitoring and managing) animals, plants and/or the environment in your community or traditional territory?
4. What comes to mind when you think of the word science or Western science?
5. Do you think Western science should have a role in helping to take care of (research, monitoring and managing) plants, animals or other parts of the environment in your community or traditional territory? Why or why not?
6. Are you familiar with times when your community included ‘this knowledge’ and Western science together for taking care of important plants, animals or other parts of the environment?
   1. What was it concerning?
   2. Who was involved?
   3. Can you tell me about the process/experience?
   4. What was it like at the beginning, middle, end?
   5. At what points/when did it go well? And why?
   6. Were there times when it went poorly and if so why?
   7. Can you foresee any difficulties with trying to consider ‘this knowledge’ and Western science together for taking care of (research, monitoring and managing) plants, animals and the environment and if so why?
   8. Do you have any suggestions of ways to overcome the challenges you just described?

* The majority of responses for this question were discussed in Menzies et al. (Menzies et al., 2022; Menzies et al., 2024).

*Some examples of bringing IK and WS together that were shared by participants*

In responses to the question about participants experience with utilizing both knowledge systems in caring for the land (e.g., oil developments, creation of wildland parks, or raising a bison calf as part of a community initiative) the role of WS was often not discussed by participants. This may be in part because the role of WS was assumed for Western endeavours or industry, or, as with details of the process of the work, because participants weren’t involved at one or several of the stages of the work. Interviewers did not probe for further information to uncover the role for each knowledge system in most instances. Here, we share a small number of examples that participants shared where IK and WS were both utilized to care for the land, where each knowledge system is clearly represented.

*“Koeye Camp is obviously one where our traditional Gvi’las were being instilled in our kids and the kids would spend time with their Raincoast crew or the weird crew talking about salmon and the species - the science aspect of the environment and the plants and animals around them...” -Anonymous*

*“I was a lead author on a paper on grizzly bear management in the Quay River to try and incorporate the science, the data that was being collected, and to put that together with the Heiltsuk knowledge and the Gvi’ilas that we have...” -William Housty*

*“Within our sturgeon project, the whole project is based on doing that. The combining and the weaving of the two knowledge systems...” -Anonymous*

*“With the Walleye hatchery, the main guy that runs it… He definitely has a lot of cultural knowledge, and he's the one that taught me about the Natural Law. He definitely is coming at it from that very traditional approach, but then at the same time, he worked for MNR [Ministry of Natural Resources] for a long time too, so he definitely has that management style as well...” -Anonymous*

*“…this fall we had noticed that the concern of water was very big. We had applied for [funds] through our IPCA [Indigenous protected and conserved areas] project …to do a bunch of water monitoring. We basically took samples throughout the water table, as well as sediment, cores, and invertebrate sampling… We really based the areas off of what the community wanted…” -Anonymous*

Literature review

**Supplementary Table S2:** List of coded questions utilized in this manuscript. Many of these questions were coded primarily for the systematic maps. Some were coded after completion of systematic map coding.

| **Items coded in n=150 case studies** |
| --- |
| Study ID |
| Coder for study, to allow cross-checks if/when needed |
| Full article citation |
| Year published |
| What is the research focus? (drop-down for research and monitoring or management) |
| What is the ecological scale of the research? (drop-down for ecosystems or species) |
| What is the research subject? Based on the previous question, list the specific ecosystem, community or species (e.g., ecosystems = grasslands, prairies, forests, woods, mountains, taiga, tundra; community = group or association of populations of two or more different species occupying the same geographical area at the same time such as bear/salmon, insect/black ash, avian cholera/eider; species = buffalo, grouse, strawberry) |
| Which themes does the research include? Select all that apply from drop-down menu. Options included: •Track and adapt to environmental/ climate change •Resource/ wildlife management •Protection of species habitat/ protected areas management •Economic development/ livelihood improvement •Document Indigenous Knowledge •Bridge IK and WS (if stated explictly as objective) •Answer scientific questions/ inform scientific research •Ensure continued access to culturally important species •Reduce deforestation •Education •Wildlife health •Ecosystem health •Track land use change •Monitoring impacts industrial activity/ development •Biodiversity conservation •Intergenerational knowledge transfer/ revitalization of Indigenous knowledge •Improving health and wellbeing •Enhance use of IK in management •Enacting IP governance and responsibilities/ territorial protection •Inform ecological restoration •NA - management study •Other: •Multiple |
| Is the study building on a previous relationship or collaboration with an Indigenous community? (dropdown: Yes, Not reported, NA-management study) |
| Are Indigenous community members included in the decision to initiate the study? (dropdown) A=Academic researchers initiate study (unless researcher is from study community and working on behalf of community needs - then code as C) M=Mutual agreement b/w community partners and researchers C=Community members/ groups initiate the study O=Other (listed under 'Notes') NR=not reported (if there is no reported data regarding this research stage - otherwise code as A if described and no community pt. is recorded) NA-management study |
| Initiating project notes - Indicate "NA, management study" if study was not research and monitoring. |
| To what level do Indigenous community members have authority in setting project objectives and research questions? Coders could choose from a drop-down menu that included:  •0 = Contractual/ no community participation (researchers make all decisions - may employ community to perform tasks; tasks are defined by the academic researcher)  •1 = Consultative (community members consulted by asking for opinions/ feedback/ recommendations - decisions made by scientists)  •2 = Collaborative (community members and researchers work together - researchers have primary authority and make decisions about/ facilitate the process for collaboration)  •3 = Collegial (researchers and community members work together - local community members have primary authority and make decisions about/ facilitate the process for collaboration)  •4 = Indigenous (community members make all decisions and have authority over all research aspects in every stage of the process)  •NR = not reported (if there is no reported data regarding this research stage - otherwise code as 0 if described and no community pt. is recorded)  •NA - management study |
| Setting project objective notes - Indicate "NA, management study" if study was not research and monitoring. |
| To what level do Indigenous community members have authority in the research design? Coders could choose from a drop-down menu that included: •0 = Contractual/ no community participation (researchers make all decisions - may employ community to perform tasks; tasks are defined by the academic researcher) •1 = Consultative (community members consulted by asking for opinions/ feedback/ recommendations - decisions made by scientists) •2 = Collaborative (community members and researchers work together - researchers have primary authority and make decisions about/ facilitate the process for collaboration) •3 = Collegial (researchers and community members work together - local community members have primary authority and make decisions about/ facilitate the process for collaboration) •4 = Indigenous (community members make all decisions and have authority over all research aspects in every stage of the process) •NR = not reported (if there is no reported data regarding this research stage - otherwise code as 0 if described and no community pt. is recorded) •NA - management study |
| Designing project notes specific to Indigenous participation - Indicate "NA, management study" if study was not research and monitoring. |
| What methodology/ methodologies are used to weave or braid knowledges? •Anishinaabe Mino-Bimaadiziwin •Ethnography •Autoethnography •Biskaabiyiyang •Community-based participatory research •Case study •Emic-Etic approach •Hunting and gathering •Self-location •Spirit-Centered •Two-Eyed Seeing •Wahkohtowin •Relational accountability •Phenomenology •Grounded theory •Experimental •Participatory modelling •Mixed methods (mixing qualitative and quantitative methodologies) •Not reported  •Other •Multiple |
| Are IK & WS braided (yes/no), and are IK and WS being used for the same and/or different things for project design (coders included both the same and different if this was the case)? Coders could choose from a drop-down list, which included the following:  •IK and WS used for the same things  •IK and WS used for different things  •No weaving practices - only IK or WS informing research design  •Other:  •Multiple |
| What are the roles for each IK and WS in research design? Coders could choose from a drop-down menu that included: Use IK as local scale expertise •IK as a source of historical/ baseline information •IK used in formulating research questions and hypotheses •Western science informing IK methods •NA - management study •Other: •Multiple |
| Designing project notes specific to braiding knowledge systems - Indicate "NA, management study" if study was not research and monitoring. |
| To what level do Indigenous community members have authority regarding the implementation of the research? Coders could choose from a drop-down menu that included: •0 = Contractual/ no community participation (researchers make all decisions - may employ community to perform tasks; tasks are defined by the academic researcher) •1 = Consultative (community members consulted by asking for opinions/ feedback/ recommendations - decisions made by scientists) •2 = Collaborative (community members and researchers work together - researchers have primary authority and make decisions about/ facilitate the process for collaboration) •3 = Collegial (researchers and community members work together - local community members have primary authority and make decisions about/ facilitate the process for collaboration) •4 = Indigenous (community members make all decisions and have authority over all research aspects in every stage of the process) •NR = not reported (if there is no reported data regarding this research stage - otherwise code as 0 if described and no community pt. is recorded) •NA - management study |
| Implementing project notes specific to Indigenous participation - Indicate "NA, management study" if study was not research and monitoring. **note that it was clarified that implementing means data collection |
| What research methods were used for Indigenous Knowledge? *Note that the knowledge type was the focus for this question. Methods were both Western social science and Indigenous, and were included in this category if they were utilized to collate IK. Coders could choose from a drop-down list, which included:  •Anishinaabe symbol-based reflection  •Ceremony  •Digital media (including participatory video or digital storytelling)  •Document review or literature review  •Fasting  •Focus groups  •GIS mapping  •Interviews (structured/semi-structured/unstructured)  •Participant observation  •Participatory mapping  •Photovoice  •Proportional piling  •Reader’s theater  •Oral history  •Survey  •Sharing circle/ talking circle  •Tobacco ties  •Workshop (including community meetings)  •Yarning (including collaborative yarning/ research topic yarning/ social yarning/ therapeutic yarning)  •Statistical analysis  •Not applicable (e.g., management and decision-making studies)  •Other:  •Multiple |
| What research methods were used for scientific data collection? Coders could choose from a drop-down list, which included: •Counts/ census data/ stock assessment •Document review or literature review •Examination of whole carcasses/ dissections •Experimental manipulation (e.g., feeding experiments) •GIS mapping •Mark-recapture studies – banding/ resight data •Natural history observations – behaviour/ reproduction/ predation events •Telemetry/ tracking devices •Tissue sampling for physiological metrics and/or genetic analysis (i.e., taking blood samples from live birds; hair samples; blubber samples; swabs for disease monitoring) •Simulation (i.e., data created from model outputs) •Not applicable (e.g., management and decision-making studies, TEK only studies, statistical analyses applied to IK) •Other: •Multiple |
| Are IK & WS braided (yes/no), and are IK and WS being used for the same and/or different things at data collection (coders included both the same and different if this was the case)? Coders could choose from a drop-down list, which included the following: •IK and WS used for the same things •IK and WS used for different things •No weaving practices - only IK or WS engaged in data collection |
| What are the roles for each IK and WS data collection? *note that this is distinct from the methods of IK and WS data collection. Coders could choose from a drop-down menu, which included:  •Use IK as local scale expertise  •IK as a source of historical/baseline information  •Western Science Informing IK methods |
| Implementing project notes specific to braiding knowledge systems. Indicate "NA, management study" if study was not research and monitoring. |
| To what level do Indigenous community members have authority regarding the analysis of the research (data analysis, interpretation, evaluation)? Coders could choose from a drop-down menu that included: •0 = Contractual/ no community participation (researchers make all decisions - may employ community to perform tasks; tasks are defined by the academic researcher) •1 = Consultative (community members consulted by asking for opinions/ feedback/ recommendations - decisions made by scientists) •2 = Collaborative (community members and researchers work together - researchers have primary authority and make decisions about/ facilitate the process for collaboration) •3 = Collegial (researchers and community members work together - local community members have primary authority and make decisions about/ facilitate the process for collaboration) •4 = Indigenous (community members make all decisions and have authority over all research aspects in every stage of the process) •NR = not reported (if there is no reported data regarding this research stage - otherwise code as 0 if described and no community pt. is recorded) •NA - management study |
| Analysis notes specific to Indigenous participation - Indicate "NA, management study" if study was not research and monitoring. |
| Are IK & WS braided (yes/no), and are IK and WS are being used for the same and/or different things at analysis and interpretation (coders included both the same and different if this was the case)? Coders could choose from a drop-down list, which included the following: •IK and WS used for the same things •IK and WS used for different things •No weaving practices - only IK or WS engaged in analysis |
| Indicate the roles for IK and WS in analysis and interpretation. Coders could choose from a drop-down menu, which included the following: •IK used in inferring impacts/ changes (i.e., mechanisms- for example for population change) •WS used in inferring impacts/ changes (i.e., mechanisms- for example for population change) •IK is discussed within the existing WS literature or vice versa •IK and WS are assigned percentage weight for decision making •NA - management study •Other: •Multiple |
| Analysis notes specific to braiding knowledge systems - Indicate "NA, management study" if study was not research and monitoring. |
| To what level do Indigenous community members have authority regarding the dissemination and/or application of the research? Coders could choose from a drop-down menu that included: •0 = Contractual/ no community participation (researchers make all decisions - may employ community to perform tasks; tasks are defined by the academic researcher) •1 = Consultative (community members consulted by asking for opinions/ feedback/ recommendations - decisions made by scientists) •2 = Collaborative (community members and researchers work together - researchers have primary authority and make decisions about/ facilitate the process for collaboration) •3 = Collegial (researchers and community members work together - local community members have primary authority and make decisions about/ facilitate the process for collaboration) •4 = Indigenous (community members make all decisions and have authority over all research aspects in every stage of the process) •NR = not reported (if there is no reported data regarding this research stage - otherwise code as 0 if described and no community pt. is recorded) •NA - management study |
| Dissemination notes - Indicate "NA, management study" if study was not research and monitoring. |
| If IK & WS were braided (yes/no) for reporting and decision making |
| Indicate the roles for IK in reporting and decision making. Coders could choose from a drop-down menu, which included the following:  •IK supporting identification of further research questions and/or management recommendations  •WS supporting identification of further research questions and/or management recommendations  •IK and WS are assigned percentage weights for decision-making  •NA - management study  •Other:  •Multiple |
| Demographics of knowledge holders - Age (dropdown: early (youth <29 yrs old), middle (30-59 yrs), late (60+ yrs), Not reported) |
| Demographics of knowledge holders - Elders (dropdown: yes, Not reported) |
| Demographics of knowledge holders - Gender (dropdown: female, male, twi-spirit, other, Not reported) |
| Are findings accessible to Indigenous community members? (drop down, including not reported and accessibility is directly addressed (e.g., findings shared with community; data available to/stored with community members; local publications produced; disseminated in local language)) |
| Are findings reported in the context of concerns, issues or interests defined by Indigenous community members? Dropdown, 0, 1 |
| How are Indigenous community members credited for their knowledge contributions and efforts? 0 = no acknowledgement 1 = acknoweldgement only (anywhere in the paper) 2 = co-authorship (contributing member of community/ community itself) NA-management study |
| Which stages were community members credited for their knowledge contributions and efforts? M1: Initiating project M2: Setting objectives M3: Designing data collecting methods M4: Collecting data M5: Analyzing and interpreting data M6: Dissemination/ application No acknowledgement NA-management study Multiple: |
| Do authors acknowledge following community protocols? (dropdown: 0=No, 1=Yes, NA-management study) |
| Do authors acknowledge that participant consent was sought? (dropdown: 0=No, 1=Yes, NA-management study) |
| Do authors acknowledge that community consent or review was sought? (dropdown: 0=No, 1=Yes, NA-management study) |
| Does the study address intellectual property rights or risks for Indigenous communities? (dropdown: 0=not reported, 1=intellectual property rights/ risks addressed, NA-management study) |
| Does the study address concerns related to dat sovereignty or information governance? (dropdown: 0=not reported, 1=some aspects of data sovereignty reported (e.g., ownership; control)) |
| Evidential notes for ethics protocols - Indicate "NA, management study" if study was not research and monitoring. |
| Does the paper acknowledge or discuss colonization and/or power? (dropdown: Yes, Not mentioned) |
| Evidential notes for colonization and/or power |
| Values discussed yes/no? |
| Evidential notes for values |

Braiding Indigenous knowledges and Western science: further information on statistical analysis

**Supplementary Table S3:** Examples of case studies belonging to the different categories in Figure 3. BIAS-K in the study ID refers to the identifiers in the systematic maps (MC = marine coastal map, FW = freshwater map, TER = terrestrial map; see references in methods). Quadrant refers to the Braiding : Engagement quadrant, where H = high, L = low, E = Engagement and B = Braiding. The numbers, from 0-1 position the study on the plot, and each axis in Figure 2 is scaled from 0-1.

| **StudyID** | **quadrant** | **Engagement** | **Braiding** | **Accessibility** | **Relevance** | **Governance** | **Previous relationship** | **Citation** |
| --- | --- | --- | --- | --- | --- | --- | --- | --- |
| BIAS-K_FW_15 | HEHB | 1 | 1 | 0 | 1 | 0 | 1 | Wheeler, B., Gilbert, M., & Rowe, S. (2012). Definition of critical summer and fall habitat for bowhead whales in the eastern Canadian Arctic. Endangered Species Research, 17, 1â€šÃ„Ã¬16. |
| BIAS-K_TER_111 | HEHB | 1 | 1 | 1 | 1 | 0 | 1 | Higdon, J.W., & Ferguson, S.H. (2011). Reports of Humpback and Minke Whales in the Hudson Bay Region, Eastern Canada Arctic. BioOne, 18(3): 370â€šÃ„Ã¬377. |
| BIAS-K_TER_173 | HEHB | 0.917 | 1 | 0 | 1 | 0 | 1 | Ostertag, S. K., Loseto, L. L., Snow, K., Lam, J., Hynes, K., & Gillman, D. V. (2018). â€šÃ„ÃºThatâ€šÃ„Ã´s how we know theyâ€šÃ„Ã´re healthyâ€šÃ„Ã¹: the inclusion of traditional ecological knowledge in beluga health monitoring in the Inuvialuit Settlement Region. Arctic Science, 4(3), 292-320. |
| BIAS-K_CM_45 | HEHB | 0.542 | 0.75 | 1 | 1 | 0 | 1 | Eckert, L.E., Ban, N.C., Frid, A., & McGreer, M. (2017). Diving back in time: Extending historical baselines for yellowed rockfish with indigenous knowledge. Aquatic Conservation: Marine and Freshwater Ecosystems, 28, 158â€šÃ„Ã¬166. |
| BIAS-K_TER_112 | HEHB | 0.542 | 0.75 | 1 | 1 | 0 | 0 | Traverse, M. J. (1999). Analyzing the Effects of the Fairford Dam on Lake St. Martin First Nation. (Master of Science Theses). University of Manitoba. Winnipeg, Manitoba, Canada. |
| BIAS-K_TER_130 | HEHB | 0.542 | 0.75 | 1 | 1 | 0 | 1 | Laidler, G. J., Hirose, T., Kapfer, M., Ikummaq, T., Joamie, E., & Elee, P. (2011). Evaluating the floe edge service: How well can SAR imagery address inuit community concerns around sea ice change and travel safety?: Evaluating the floe edge service. The Canadian Geographer, 55(1), 91-107. |
| BIAS-K_TER_116 | HELB | 0.667 | 0.333 | 1 | 1 | 0 | 0 | Housty, W. G., Noson, A., Scoville, G. W., Boulanger, J., Jeo, R. M., Darimont, C. T., & Filardi, C. E. (2014). Grizzly bear monitoring by the Heiltsuk people as a crucible for First Nation conservation practice. Ecology and Society, 19(2). |
| BIAS-K_TER_121 | LEHB | 0 | 1 | 0 | 1 | 0 | 0 | Sanderson, D., Picketts, I. M., DâˆšÂ©ry, S. J., Fell, B., Baker, S., Leeâ€šÃ„ÃªJohnson, E., & Auger, M. (2015). Climate change and water at Stellat'en First Nation, British Columbia, Canada: Insights from western science and traditional knowledge. The Canadian Geographer/Le GâˆšÂ©ographe canadien, 59(2), 136-150. |
| BIAS-K_TER_153 | LEHB | 0 | 1 | 0 | 0 | 0 | 0 | Ritts, M., Gage, S. H., Picard, C. R., Dundas, E., & Dundas, S. (2016). Collaborative research praxis to establish baseline ecoacoustics conditions in Gitgaâ€šÃ„Ã´at Territory. Global Ecology and Conservation, 7, 25-38. |
| BIAS-K_TER_44 | LEHB | 0 | 1 | 0 | 1 | 0 | 0 | Parlee, B. L., Sandlos, J., & Natcher, D. C. (2018). Undermining subsistence: Barren-ground caribou in a â€šÃ„Ãºtragedy of open accessâ€šÃ„Ã¹. Science Advances, 4(2), e1701611. |
| BIAS-K_CM_6 | LEHB | 0.5 | 0.75 | 1 | 1 | 1 | 1 | Sable, T., Howell, G., Wilson, D., & Penashue, P. (2007). The Ashkui project: Linking western science and Innu environmental knowledge in creating a sustainable environment. In P. Sillitoe (Ed.,) Local science vs. global science: approaches to indigenous knowledge in international development (pp. 109-127). New York and Oxford: Berghahn Books. |
| BIAS-K_TER_11 | LEHB | 0.5 | 0.75 | 1 | 1 | 1 | 1 | Trusler, S., & Johnson, L. M. (2008). â€šÃ„ÃºBerry patchâ€šÃ„Ã¹ as a kind of placeâ€šÃ„Ã®The ethnoecology of black huckleberry in northwestern Canada. Human Ecology, 36(4), 553-568. |
| BIAS-K_TER_96 | LEHB | 0.5 | 0.75 | 1 | 0 | 0 | 0 | Mallory, M., Fontaine, A., & Akearok, J. (2013). Status of Harlequin Duck Histrionicus histrionicus on Baffin Island, Nunavut, Canada.. Wildfowl, 54(54), 95-102. |
| BIAS-K_TER_140 | LELB | 0 | 0.25 | 0 | 0 | 0 | 0 | Henri, D. A., Martinez-Levasseur, L. M., Weetaltuk, S., Mallory, M. L., Gilchrist, H. G., & Jean-Gagnon, F. (2020). Inuit knowledge of Arctic Terns (Sterna paradisaea) and perspectives on declining abundance in southeastern Hudson Bay, Canada. PloS one, 15(11), e0242193. |
| BIAS-K_CM_10 | LELB | 0.042 | 0.25 | 0 | 0 | 0 | 0 | DeRoy, B. C., Brown, V., Service, C. N., Leclerc, M., Bone, C., McKechnie, I., & Darimont, C. T. (2021). Combining high-resolution remotely sensed data with local and Indigenous Knowledge to model the landscape suitability of culturally modified trees: biocultural stewardship in Kitasoo/Xaiâ€šÃ„Ã´xais Territory. FACETS, 6(1), 465-489. |
| BIAS-K_CM_2 | LELB | 0.083 | 0.25 | 0 | 0 | 0 | 1 | York, J., Dowsley, M., Cornwell, A., Kuc, M., & Taylor, M. (2016). Demographic and traditional knowledge perspectives on the current status of Canadian polar bear subpopulations. Ecology and evolution, 6(9), 2897-2924. |
| BIAS-K_TER_84 | LELB | 0.208 | 0.5 | 1 | 0 | 0 | 0 | Polfus, J.L., Heinemeyer, K., Hebblewhite, M., & Taku River Tlingit First Nation (2014). Comparing traditional ecological knowledge and western science woodland caribou habitat models. The Journal of Wildlife Management 78(1), 112-121. |
| BIAS-K_TER_160 | LELB | 0.292 | 0.5 | 1 | 1 | 0 | 0 | Gomes, T. C. (2012). Restoring Tl'châˆšÂ©s: an ethnoecological restoration study in Chatham Islands, British Columbia, Canada [Doctoral dissertation, University of Victoria]. |
| BIAS-K_TER_71 | LELB | 0.375 | 0.5 | 0 | 1 | 1 | 1 | Tremblay, M., Furgal, C., LarrivâˆšÂ©e, C., Annanack, T., Tookalook, P., Qiisik, M., ... & Barrett, M. (2008). Climate change in northern Quebec: Adaptation strategies from community-based research. Arctic, 27-34. |


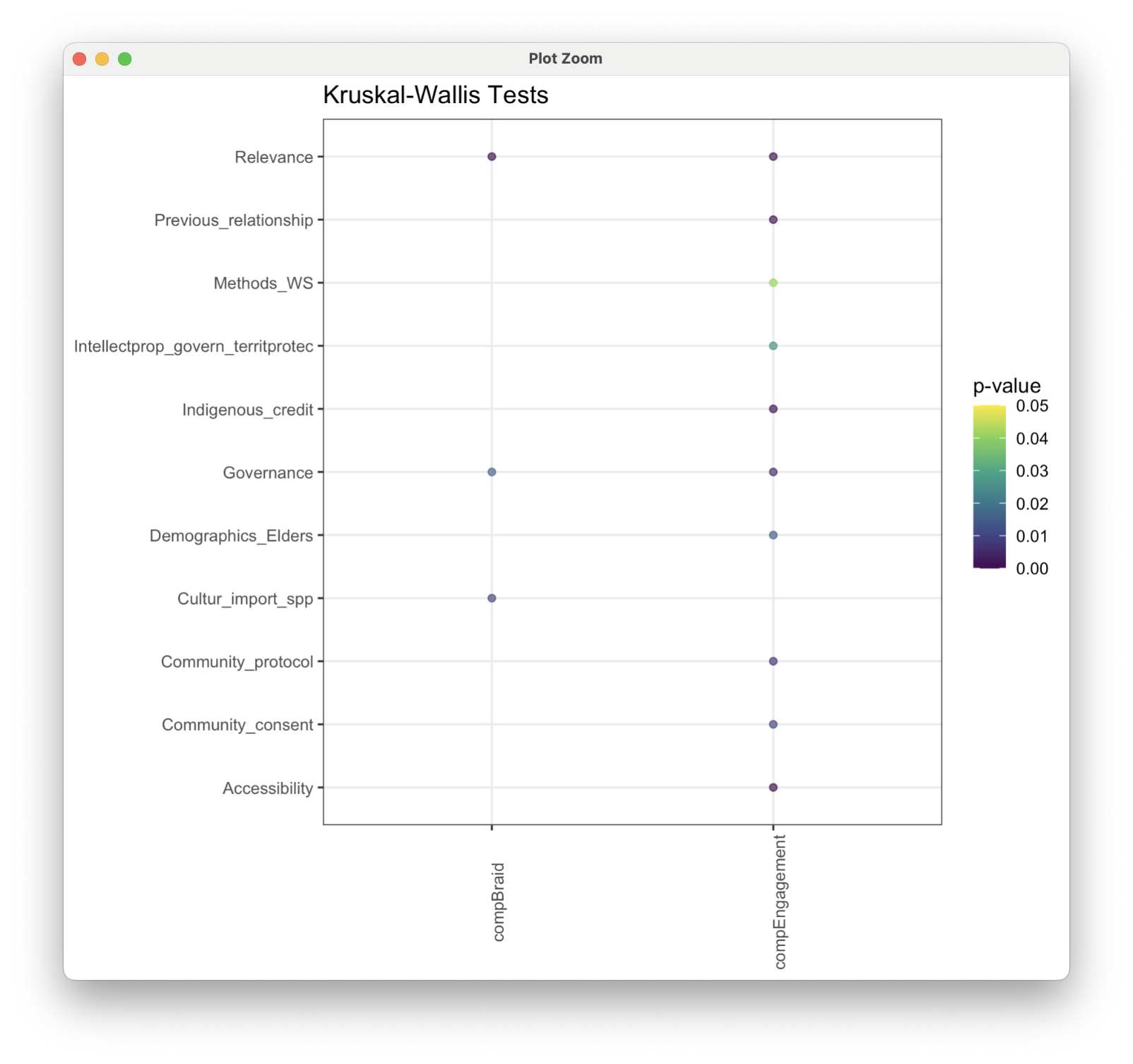


**Supplementary Figure S1**: Kruskal-Wallis test showing the proxy variables for the pillars and priorities that were correlated with the composite scores for each the number of project stages at which both IK and WS were used across the stages of a project (compBraiding) and the level of engagement with Indigenous communities across projects (compEngagement) in the systematic map studies.


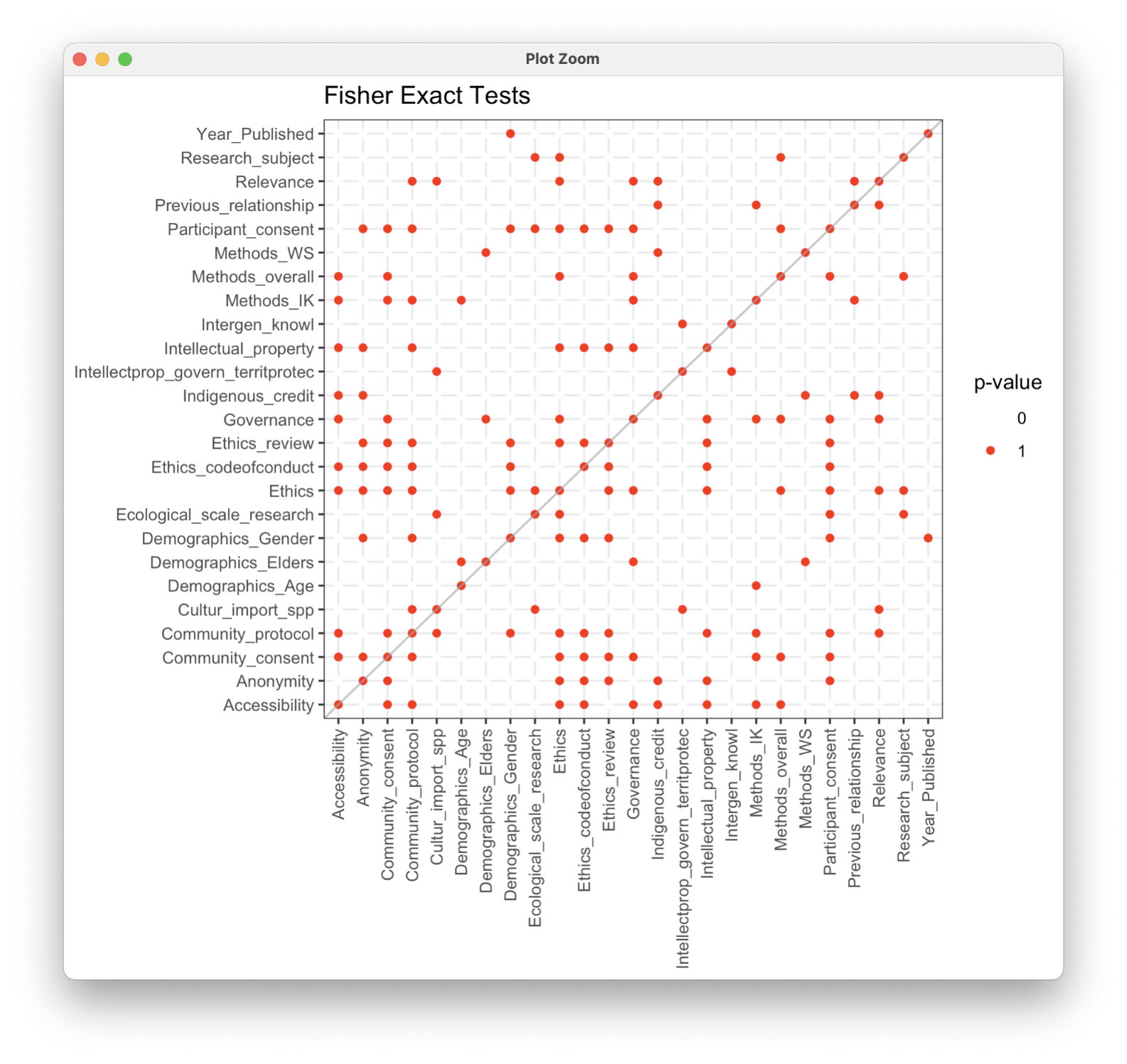


**Supplementary Figure S2:** Visualization of Fisher’s Exact Tests showing which variables were correlated (red indicates correlation).


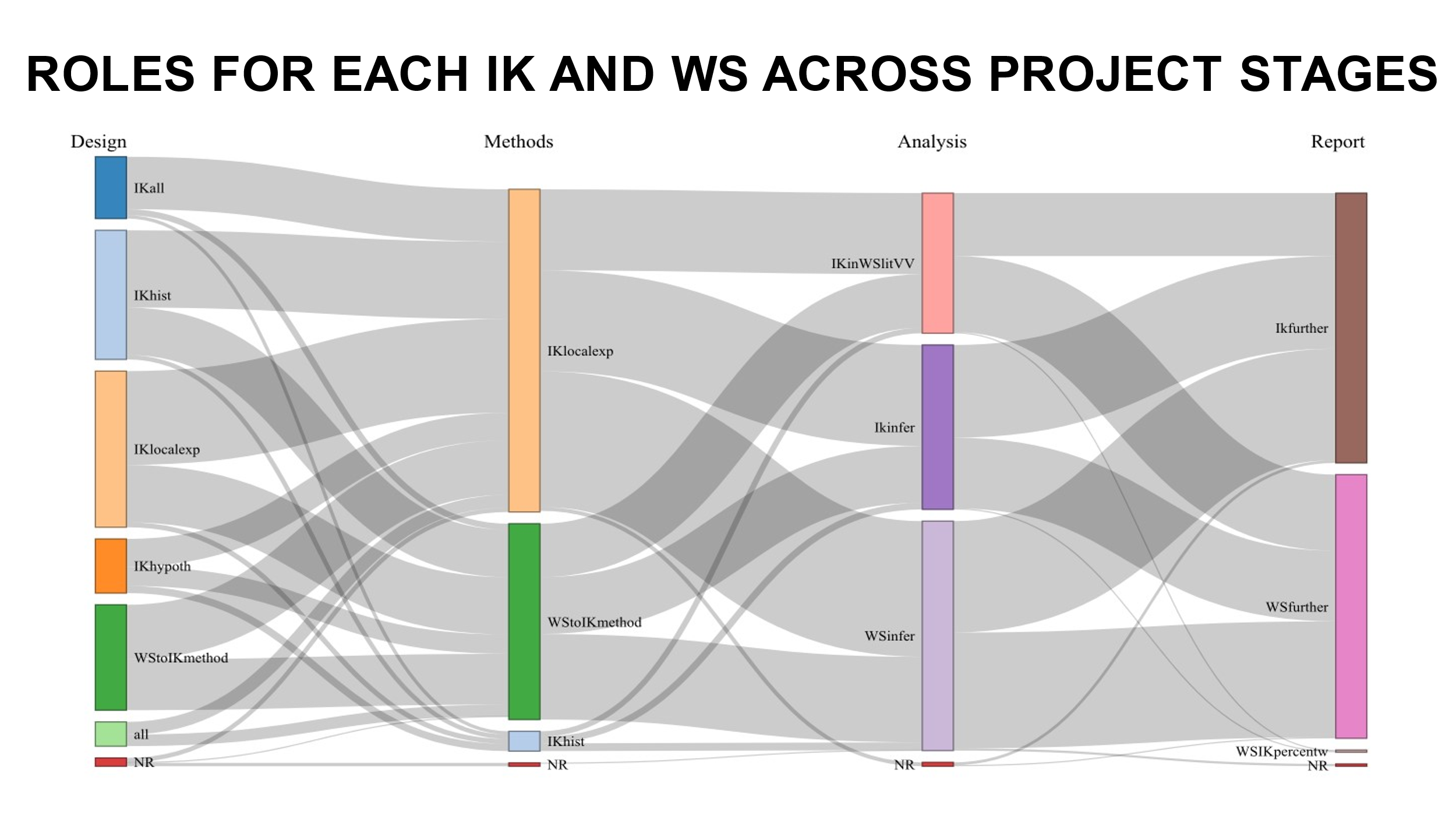


**Supplementary Figure S3:** Sankey diagram showing the roles that each IK and WS had across all project stages (design, data collection, analysis, reporting and decision making). Note that one study could be represented multiple times in a given column. we include fully written out codes here:

- IK all: Use IK as local scale expertise; IK&WS co-design equally; IK as a source of historical/baseline information; IK used in formulating research questions and hypotheses; Western science informing IK methods
- IKlocalexp_IKhypoth: Use IK as local scale expertise and IK used in formulating research questions and hypotheses
- IKlocalexp: Use IK as local scale expertise
  - IKlocalexp_WStoIKmethod: Use IK as local scale expertise and WS informing IK methods
- IKinfer_IKinWSlitVV: IK used in inferring impacts/ changes (i.e., mechanisms- for example for population change) and IK is discussed within the existing WS literature or vice versa
  - WSinfer_IKinfer_IKinWSlitVV: both WS and IK are used in inferring impacts/ changes (i.e., mechanisms- for example for population change) and IK is discussed within the existing WS literature or vice versa
- WSinfer_IKinWSlitVV: WS used in inferring impacts/ changes (i.e., mechanisms- for example for population change) and IK is discussed within the existing WS literature or vice versa
- WSinfer: IK used in inferring impacts/ changes (i.e., mechanisms- for example for population change)
- WSinfer_IKinfer: both WS and IK are used in inferring impacts/ changes (i.e., mechanisms- for example for population change)
- IKfurther: IK supporting identification of further research questions and/or management recommendations
  - WSfurther_IKfurther: Both WS and IK supporting identification of further research questions and/or management recommendations
  - NR: not reported

**Supplementary Table S4:** Extended table of the roles for braiding IK and WS, including examples. The references listed as examples in this table are not in the literature cited list in this manuscript unless they were cited elsewhere in the main text of the manuscript. However, they can be found in the databases associated with each of the systematic maps the using the “BIAS-K” number.

| **Abbreviation** | **Full Name** | **Examples** |
| --- | --- | --- |
| **WStoIKmethod** | Western science informing IK methods | Both WS and IK considered in project design: "In fall 2008/winter 2009, DFO’s Oceans Program conducted meetings in Nunavut with the Regional Inuit Associations (RIAs), Nunavut Tunngavik Inc. (NTI), Nunavut Wildlife Management Board (NWMB) and Government of Nunavut (GN) to consider areas in Nunavut that might be considered for Marine Protected Area designation... DFO Science was then asked to identify areas which could be considered as being ecologically or biologically significant within this study area... (p.6)." ; "A science advisory meeting was held in June 2009 to assess the available scientific knowledge for northern Foxe Basin to determine whether one or more locations/areas within it would qualify as an EBSA. Participants also considered available published traditional/local knowledge. This document presents the information peer reviewed during the meeting... (p.7)." BIAS-K_CM_41 (Paulic et al. 2014)  "We estimated trends in large abalone densities… using density-independent exponential population growth models fit to TK and WSK data using Bayesian methods… Only WSK data were modeled for small abalone <70 mm… due to few TK data points .Each TK observation represented average abalone density by site by decade. Each WSK observation represented total number of abalone per site per year... (p.3)." BIAS-K_CM_64 (Lee et al. 2018) |
| **IKlocalexp** | Use IK as local scale expertise | During an earlier phase, the lead author spent several months in the field conducting semi- structured interviews with 33 Indigenous knowledge holders as well as 19 decision-makers at local and regional levels to document and understand community vulnerability to environmental change. This, combined with intensive participant observation, provided a substantive contextual background and clearly identified the two primary drivers of environmental change (BIAS-K_FW_32) (Wesche et al. 2014) |
| **WSinfer** | WS used in inferring impacts// changes (i.e., mechanisms- for example for population change) | Western statistical analysis was done on all data collected from interviews and field observations: Hunters impressions of body condition recorded in the field were compared with those provided in interviews. For both years, the distribution of field impressions for hunters who harvested (a) less than (or equal to) or (b) more than the average number of caribou was assessed using Kolmogorov-Smirnov two-sample test. The distribution of hunters impressions in relation to hunting experience was also assessed. Differences in body condition of adult female caribou harvested in 2001 around Lutsel K and Austin Lake (50 km southeast of the community) were compared using a Mann-Whitney U-Test. The body condition of mature cows harvested in February, March, and April was also compared using a Kruskal-Wallis one-way analysis of variance (BIAS-K_TER_148) (Lyver et al. 2005) |
| **IKinWSlitVV** | IK is discussed within the existing WS literature or vice versa | IK analysed based on consistency with existing WS: The natural history descriptions of the species habits were consistent with the limited information available in the scientific literature, suggesting that Inuit are astute observers of their environment, the LEK presented to us was undoubtedly a recounting of the report published by Bray (1943)... Both LEK and scientific investigation indicate that the climate, in terms of sea ice conditions, is changing in this area... LEK information has been used to assess population status... Despite some inconsistencies in LEK on population trends, we interpreted the overall comments as indicative of population declines near all three communities. The LEK was strongly supported by the observations of government officials and ship personnel (BIAS-K_CM_9) (Mallory et al. 2003) |
| **WSfurther** | WS supporting identification of further research questions and/or management recommendations | Although we acknowledged that definitive scientific diagnosis of avian cholera was required by laboratory analyses, this TEK study supported the hypothesis that current and ongoing outbreaks of avian cholera are recent in the eastern Canadian Arctic, having been first detected by Inuit in the mid-2000s...this important source of knowledge to be meaningfully included and combined with scientific information in joint avian disease and wildlife health monitoring initiatives (BIAS-K_CM_8) (Henri et al. 2018) |
| **IKall** | Use IK as local scale expertise, IK as a source of historical/baseline information, IK used in formulating research questions and hypotheses | The local expert fishers in Sachs Harbour and Ulukhaktok were directly involved in research design and determination of study locations and environmental and ecological parameters for scientific sampling. Local assistants were trained in scientific sampling methods and worked with the researcher throughout the project to sample all fish habitat parameters and to collect data from fish caught in scientific nets and local subsistence catches (BIAS-K_FW_33) (Knopp et al. 2012) |
| **IKinfer** | IK used in inferring impacts/ changes (i.e., mechanisms- for example for population change) | The research findings were reviewed with community members thus providing a check on the extent to which the researchers captured community understanding of change in ice exposures and key drivers of change. The use of multiple translations, and the cross referencing of narratives obtained in the interviews with participant observation and secondary sources enabled the information obtained to be checked to assess consistency and credibility in the findings (BIAS-K_TER_78) (Ford et al. 2008) |
| **IKfurther** | IK supporting identification of further research questions and/or management recommendations | TEK that reflected values about the environment... and the Inuvialuit knowledge system... were reviewed by the researchers to provide recommendations on how they may support beluga monitoring and management. The inclusion of Inuvialuit in the FJMC ensures their integration in the functions and decisions pertaining to fisheries management in the ISR... (BIAS-K_CM_45) (Ostertag et al. 2018) |
| **IKhypoth** | IK used in formulating research questions and hypotheses | Interviews were employed to document Cree knowledge related to moose, forestry impacts and needs pertaining to forest management. Telemetry was employed to gather scientific information on moose habitat use and scientific methods were informed by findings from interview with Cree participants (BIAS-K_TER_178) (Jacqmain et al. 2006) |
| **IKhist** | IK as a source of historical/baseline information | Open-ended, semi-formal, and informal interviews were conducted with indigenous consultants familiar with the plant and/or habitat of interest. Traditional knowledge of appropriate restoration sites was used in a field experiment to re-establish sweetgrass in an area from which it is believed to have been extirpated. Traditional knowledge of anthropogenic burning was used to reintroduce fire in low-elevation beargrass habitats to manage both the resource and its environment. By incorporating traditional knowledge with published information on sweetgrass biology, it was found that two potential factors influencing its population in cultural gathering sites are unsustainable harvesting and the absence of controlled burns (BIAS-K_TER_177) (Shebitz 2005) |
| **all** | Use IK as local scale expertise, IK as a source of historical/baseline information, IK used in formulating research questions and hypotheses, Western science informing IK methods | The methodology for this research involved the integration of two emerging knowledge systems relevant to monitoring salmon and salmon fisheries: quantitative methods creating time series graphs from annual escapement... and native harvest statistics; and social science methods yielding indigenous knowledge and perspectives of the Goldstream salmon fisheries. An interdisciplinary research approach, blending science and social science methods was therefore undertaken to address the question of how escapement counts contribute to Goldstream salmon monitoring protocols (BIAS-K_FW_49) (Paul 2006) |
| **WSIKpercentw** | IK and WS are assigned percentage weights for decision-making | The one example where percentage weight was coded uses percentage weight is the primary means to communicate IK and WS information. as percentages (BIAS-K_TER_101) (Herman-Mercer et al. (2020). The code was derived however based on a study that was ultimately excluded from this study, where IK and WS were assigned percentage weights in a decision-making matrix. |
